# How the credit assignment problems in motor control could be solved after the cerebellum predicts increases in error

**DOI:** 10.3389/fncom.2015.00039

**Published:** 2015-03-24

**Authors:** Sergio O. Verduzco-Flores, Randall C. O'Reilly

**Affiliations:** Computational Cognitive Neuroscience Laboratory, Department of Psychology and Neuroscience, University of Colorado BoulderBoulder, CO, USA

**Keywords:** cerebellum, reaching, equilibrium point, motor learning, complex spikes

## Abstract

We present a cerebellar architecture with two main characteristics. The first one is that complex spikes respond to increases in sensory errors. The second one is that cerebellar modules associate particular contexts where errors have increased in the past with corrective commands that stop the increase in error. We analyze our architecture formally and computationally for the case of reaching in a 3D environment. In the case of motor control, we show that there are synergies of this architecture with the Equilibrium-Point hypothesis, leading to novel ways to solve the motor error and distal learning problems. In particular, the presence of desired equilibrium lengths for muscles provides a way to know when the error is increasing, and which corrections to apply. In the context of Threshold Control Theory and Perceptual Control Theory we show how to extend our model so it implements anticipative corrections in cascade control systems that span from muscle contractions to cognitive operations.

## 1. Introduction

The anatomy of the cerebellum presents a set of well established and striking facts (Eccles et al., 1967; Ito, 2006), which have inspired a variety of functional theories over the years. The cerebellum receives two main input sources, the mossy fibers and the climbing fibers. The mossy fibers convey a vast amount of afferent and efferent information, and synapse onto granule cells, Golgi cells, and neurons of the deep cerebellar nuclei. Granule cells exist in very large numbers, and could be considered the input layer of the cerebellum; they send axons that bifurcate in the cerebellar cortex, called parallel fibers, innervating Purkinje cells and molecular layer interneurons. Purkinje cells have intricate dendritic arbors with about 150,000 parallel fiber connections. On the other hand, each Purkinje cell receives a single climbing fiber that can provide thousands of synapses. Activation of a climbing fiber reliably causes a sequence of tightly coupled calcium spikes, known as a complex spike. In contrast, simple spikes are the action potentials tonically produced by Purkinje cells, modulated by parallel fiber inputs and feedforward inhibition from molecular layer interneurons. The sole output from the cerebellar cortex is constituted by the Purkinje cell axons, which send inhibitory projections to the deep cerebellar nuclei and to the vestibulum. Cells in the deep cerebellar nuclei can send projections to diverse targets, such as the brainstem, the thalamus, the spinal cord, and the inferior olivary nucleus. The inferior olivary nucleus is the origin of climbing fibers, which are the axons of electrotonically-coupled olivary cells that experience subthreshold oscillations in their membrane potential.

There is a prevailing view that the cerebellum is organized into modular circuits that perform similar computations. Sagittal regions of Purkinje cells called microzones receive climbing fibers from a cluster of coupled olivary neurons, and tend to be activated by the same functional stimuli. Purkinje cells in a microzone project to the same group of cells in the cerebellar nuclei, which in turn send inhibitory projections to the olivary neurons that innervate the microzone. A microzone together with its associated cerebellar nuclear cells is called a microcomplex, which together with its associated olivary cells constitutes an olivo-cerebellar module.

In one of the first and most influential theories about cerebellar function, developed by a succession of researchers (Marr, 1969; Albus, 1971; Ito et al., 1982), the convergence of mossy fibers (which carry sensory and motor signals into the cerebellum) onto Purkinje cells supports pattern recognition in a manner similar to a perceptron. This pattern recognition capacity is used to improve motor control, and the Marr-Albus-Ito hypothesis states that the other major cerebellar input, the climbing fibers, provide a training signal that, thanks to conjunctive LTD on the parallel fiber synapses into Purkinje cells, allows for the right patterns to be selected. Conjuctive LTD (Long-Term Depression) reduces the strength of parallel fiber synapses when they happen to be active at the same time as climbing fiber inputs. Within this general framework, a persistent challenge comes in determining what the right patterns are, and how they are used to improve motor control.

One common trend for cerebellar models of motor control is to assume that the cerebellum is involved in providing anticipative corrections to performance errors (Manto et al., 2012), and that this is done by forming internal models of the controlled objects (Wolpert98,Ebner13). Forward models take as inputs a command and a current state, returning the consequences of that command, often in the form of a predicted state. Inverse models take as their input a desired state and a current state, returning the commands required to reach the desired state. Adaptive learning in the cerebellum is often assumed to involve using error signals to learn these types of internal models. It should be noted that some computational elements (such as adaptive filters), which could be implemented by cerebellar microzones, can in principle learn to implement either a forward or an inverse model depending on its input/output connections and on the nature of its error signal (Porrill et al., 2013).

**Figure 1:**
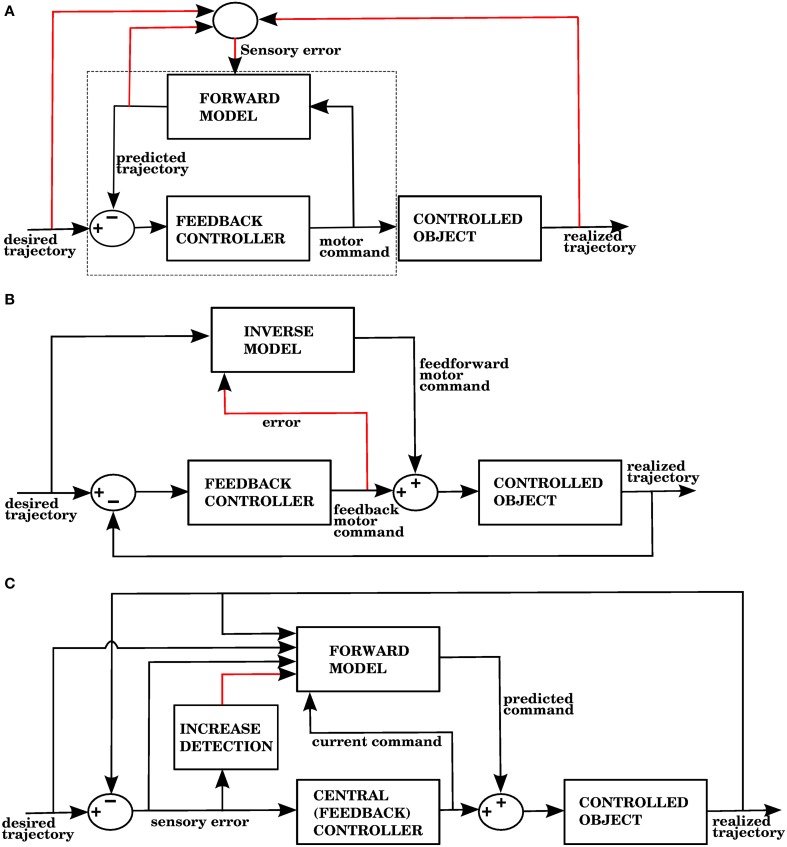
**(A)** The recurrent architecture (Porrill and Dean, 2007) uses a forward model as the adaptive element in a controller. This forward model learns to predict the response of the controlled object to the motor commands, using an error that considers the difference between the predicted trajectory and the realized trajectory. Notice that the elements inside the dashed rectangle constitute an adaptive inverse model of the controlled object. Red lines indicate signals used for training of the forward model. Based on Figure 1A of Ito (2013). **(B)** Use of an inverse model to improve the performance of a feedback controller using the feedback error learning scheme of Kawato and Gomi (1992). The output of the feedback controller is used to approximate the error in the motor command, so the inverse model can be trained. The red line indicates the learning signal. **(C)** A forward model proposed in this paper is used to improve the performance of a central controller. The forward model associates a context consisting of a variety of sensory and motor signals (black arrows entering from the left) with a command produced by the controller (black arrow entering from below). The context will be associated with future controller commands whenever the sensory error increases, indicated by the red line. Notice that while the forward model in **(A)** predicts the response of the controlled object, the forward model in **(C)** predicts the response of the central controller. In the Section 3, model 1 corresponds to this architecture.

The error signal required by a forward model is a sensory error, which consists of the difference between the desired sensory state (e.g., a hand trajectory) and the perceived sensory state. In contrast, inverse models require a motor error signal that indicates the difference between a given command and the command that would have produced the desired outcome. Figures 1A,B shows two well known proposed architectures that allow the cerebellum to use forward and inverse models to reduce performance errors, respectively called the recurrent architecture, and feedback error learning. A recent review (Ito, 2013) examined the signal contents of climbing fibers for different cerebellar circuits, and found that both sensory and motor errors might be present, bringing the possibility of having both forward and inverse models in the cerebellum.

Inverse models in the cerebellum present some difficulties. The first one is known as the motor error problem, and consists on the requirement that the climbing fibers carry an unobservable motor error rather than the observed sensory error signal. This creates difficulties when applying them to the control of complex plants (Porrill et al., 2013). A second difficulty is the evidence that simple spikes in Purkinje cells are consistent with a forward model, but probably not with an inverse model (Ebner and Pasalar, 2008). Although climbing fiber may carry information about motor errors, most studies seem to find correlations with sensory signals and sensory errors (e.g., Ghelarducci et al., 1975; Stone and Lisberger, 1986; Ekerot et al., 1991; Yanagihara and Udo, 1994; Kitazawa et al., 1998; Simpson et al., 2002).

There are two other problems that must be addressed by cerebellar models that form internal models, whether forward or inverse (Porrill and Dean, 2007). The distal error problem happens when we use output errors (such as sensory signals) to train the internal parameters of a neural network. Backpropagation is a common—although biologically implausible—way to deal with this problem. In reaching, the distal error problem can be conceived in terms of knowing which muscles to stimulate if you want the hand to move in a certain direction. The nature of the distal error problem is the same one as that of the motor error problem, since they both are credit assignment problems. After an error is made, the credit assignment problem consists in knowing which control signals contributed to the error, and which ones can reduce it. The redundancy problem happens when a set of commands lead to the same outcome, leading to incorrect generalizations when that set is non convex. One common setting where the redundancy problem arises is in reaching. The human arm, including the shoulder and elbow joints has 5° of freedom (without considering shoulder translation), allowing many joint configurations that place the hand in the same location.

The recurrent architecture of Figure 1A, and the feedback error learning scheme of Figure 1B are shown here because they present two different ways of addressing the motor error and redundancy problems. The recurrent architecture is trained with sensory error, so the motor error problem is not an issue; moreover, this architecture receives motor commands as its input, so it doesn't have to solve the redundancy problem. Feedback error learning approximates the motor error by using the output of a feedback controller. The feedback controller thus acts as a transformation from sensory error into motor error. If the feedback controller can properly handle redundancy, then so will the inverse model that it trains.

In this paper we propose a new cerebellar architecture that successfully addresses the motor error problem, the distal learning problem, and the redundancy problem. This architecture is specified at an abstract level, and consists of descriptions of the inputs and outputs to cerebellar modules, the content of climbing fiber signals, and the nature of the computations performed by the cerebellar microzone.

In our architecture, the role of the cerebellum is to provide anticipative corrections to the commands issued by a central controller, and we explore 4 variations on how to associate a predicted increase in error with the corrective motor commands. For example, in the first version of our architecture (called model 1 in the Section 3), shown in Figure 1C, these corrections are learned by associating the sensory/motor context shortly before an error with the corrective response issued by the central controller shortly afterwards. We thus propose that the cerebellar inputs carried by mossy fiber signals consist of all sensory and motor signals that can be used to predict a future state. The cerebellar output could be a predicted set of motor commands similar to a correction issued by the central controller in the past. The climbing fiber activity rises in response to an *increase* of an error measure over time, not to instantaneous error values. Cerebellar microzones act to predict an increase in error, and this prediction is then associated with a correction. For example, in our first variation of the architecture (model 1), particular sensory/motor contexts are associated with a response by the central controller happening shortly after an increase in the climbing fiber activity. This is consistent with many models based on the Marr-Albus-Ito framework. If a bank of filters (presumably arising from computations in the granule cell layer) are placed in the inputs, then this associator becomes functionally similar to adaptive filter models commonly found in cerebellum literature (Fujita, 1982; Dean and Porrill, 2008). Those models usually assume that mossy fiber inputs correlated with climbing fiber activity cause a decrease in the firing rate of Purkinje cells because of conjunctive LTD, leading to an increase in firing rate at the cerebellar or vestibular nuclei. This could be conceived as associating a particular pattern of mossy fiber inputs with a response in cerebellar nuclei, with the input filters giving the system the ability to recognize certain temporal patterns.

We explore the ideas of our cerebellar architecture by implementing it in computational and mathematical models of reaching in 3D space. We chose this task because it presents challenges that should be addressed by cerebellar models, namely distal learning, redundancy, and timing. There is a tendency for studies of the cerebellum in motor control to model problems where the error signal is 1-dimensional, thus hiding the difficulties of distal learning and redundancy. For example, the distance between the hand and the target is a 3-dimensional error, but it can be decomposed into 1-dimensional errors (left-right, up-down, forward-backward). In a different example, for 2D reaching with a planar arm joint-angle errors can be used, so the error signal already implies what the right correction is. In the present study we try to break away from this tendency.

In the context of reaching, the idea that the cerebellum could function by anticipatively applying the same corrections as the central controller raises valid concerns about stability. We address these concerns by showing that if the central controller acts like a force always pointing at the target, and whose magnitude depends only in the distance between the hand and the target, then an idealized implementation of our cerebellar architecture will necessarily reduce the energy of the system, resulting in smaller amplitude for the oscillations, and less angular momentum. The idealized implementation of the architecture thus yields sufficient conditions for its successful application. This result is presented in the Supplementary Material.

In addition to our mathematical model, we implemented four computational models of a 3D reaching task embodying simple variations of our proposed architecture. The central controller in the four models uses an extension of the Equilibrium-point hypothesis (Feldman and Levin, 2009), described in the Section 2. The presence of equilibrium points permits ways of addressing the motor error problem different than using stored copies of efferent commands from the central controller, and ways of detecting errors different than visually monitoring the distance between the hand and the target. Our four models thus explore variations of the architecture, in which either the learning signal or the corrections are generated using proprioceptive signals from muscles. For these models the controlled plant is a 4 DOF arm actuated by 11 Hill-type muscles. The cerebellar module associates contexts, represented by radial basis functions in the space of afferent and motor signals with corrective motor commands. These associations between contexts and motor responses happen whenever a learning signal is received, which happens when there is an increase of the error.

As mentioned above, we use two types of errors in our computational models. The first type of error is the distance between the hand and the target, which proves to be sufficient to obtain predictive corrections. By virtue of using the equilibrium-point hypothesis in the central controller we can alternatively use a second type of error signal generated for individual muscles that extend when they should be contracting. This allows the cerebellum to perform anticipative corrections in a complex multidimensional task like reaching using learning signals that arise from 1-dimensional systems. This learning mechanism can trivially be extended to serial cascades of feedback control systems, such as those posited by Perceptual Control Theory (Powers, 1973, 2005) and Threshold Control Theory (Feldman and Levin, 2009; Latash et al., 2010), allowing the cerebellum to perform corrections at various levels of a hierarchical organization spanning from individual muscle contractions to complex cognitive operations. We elaborate on this in the Discussion.

## 2. Materials and methods

### 2.1. Physical simulation of the arm

**Figure 2:**
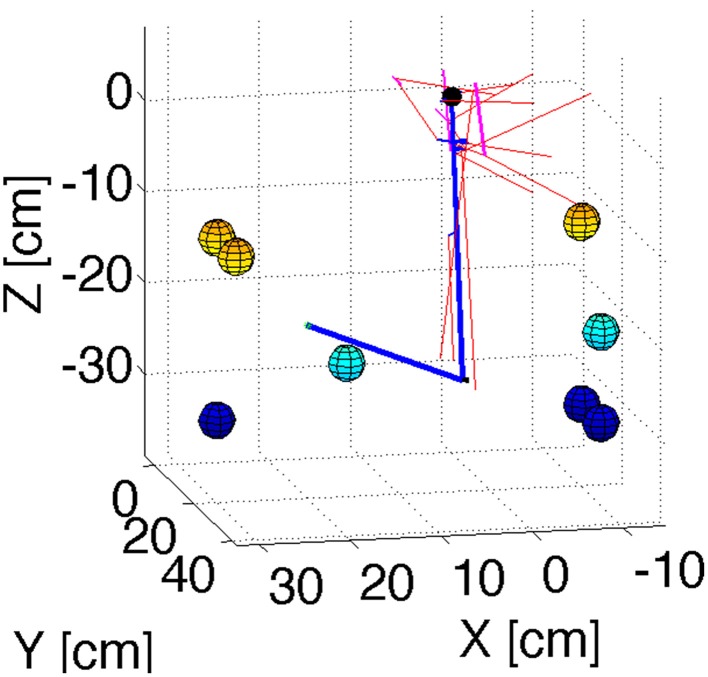
**Geometry of the arm model**. Blue lines represent the upper arm and forearm, with the small black sphere representing the shoulder. Red lines represent muscles. Cyan lines are bending lines. The colored spheres (with color representing their position along the Z axis) show the location of the targets used in the reaching simulations. The coordinates of these targets are in Table 1.

In order to test the principles of our cerebellar model in 3D reaching tasks we created a detailed mechanical simulation of a human arm. Our arm model contains a shoulder joint with 3° of rotational freedom, and an elbow joint with one degree of rotational freedom. Inertia tensors for the arm, forearm, and hand were created assuming a cylindrical geometry with size and mass typical of human subjects. The actuators consist of 11 composite muscles that represent the main muscle groups of the human arm (Figure 2). Some of these muscles wrap around “bending lines,” which are used to model the curved shape of real muscles as they wrap around bones and other tissue. The force that each muscle produces in response to a stimulus comes from a Hill-type model used previously with equilibrium point controllers (Gribble et al., 1998). The mechanical simulation was implemented in SimMechanics, which is part of the Matlab/Simulink software package (http://www.mathworks.com/), release 2012b. Source code is available from the first author upon request.

The coordinate for the targets used in our test reaches are shown in Table 1.

**Table 1:** **Coordinates used for the targets in the test reaches**.

	**X [cm]**	**Y [cm]**	**Z [cm]**
Target 1	−10	20	−30
Target 2	−10	20	−10
Target 3	−10	30	−30
Target 4	−10	30	−20
Target 5	30	20	−30
Target 6	30	20	−10
Target 7	20	40	−20
Target 8	30	30	−10

*The origin is at the shoulder. The X axis points to the right, the Y axis to the front, and the Z axis upwards*.

### 2.2. Central controller

The central controller we use to perform reaching is a modified version of Threshold Control Theory (TCT, Feldman and Levin, 2009). TCT is an extension of a biological control scheme known as the Equilibrium Point (EP) hypothesis. The lambda version of the EP hypothesis states that the control signals used in the spinal cord to drive skeletal muscles consist of a group of muscle lengths known lambda values. When the length of a muscle exceeds its lambda value it contracts, so that a set of lambda values will lead the body (or in our case, the arm) to acquire an equilibrium position. The muscle lengths at the equilibrium position may or may not be equal to their lambda values. Also, notice that given a set of lambda values there is a unique position that the limb will acquire, because the viscoelastic properties of the muscles will lead the joint to adopt the configuration minimizing its potential energy. In this paper the control signals arriving at the spinal cord to specify threshold lengths for muscle activation are called target lengths.

Considering that the velocity of a muscle's extension-contraction is represented in spindle afferents (Lennerstrand, 1968; Lennerstrand and Thoden, 1968; Dimitriou and Edin, 2008, 2010), the argument made for lengths in the EP hypothesis could be modified to hypothesize threshold velocities being the control signals at the spinal cord level, and threshold lengths being used at a higher level, modulating the threshold velocities. Such a two level control system is inspired by the hierarchical organization found in TCT and in Perceptual Control Theory (Powers, 1973, 2005), and is capable of stabilizing oscillations with far more success than pure proportional control. In general, it is hard to stabilize movement without velocity information, so this factor has been introduced in equilibrium-point controllers (de Lussanet et al., 2002; Lan et al., 2011). As in TCT, we assume that the forces are generated at the level of the spinal cord, similarly to the stretch reflex, and we assume a proprioceptive delay of 25 ms.

**Figure 3:**
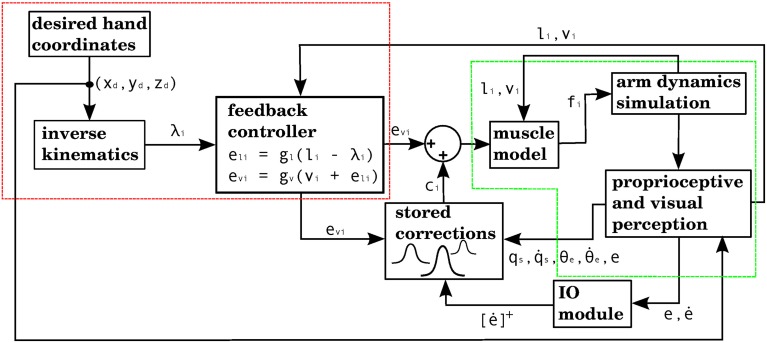
**Block diagram corresponding to the computational implementation of our architecture in Matlab when using visual errors**. λ_*i*_ is the target length for muscle *i*. *e*_li_ and *e*_vi_ are respectively the length and velocity errors for the *i*-th muscle. *c_i_* is the cerebellar correction applied to muscle *i*. *f_i_* is the force vector applied on the insertion points of muscle *i* as a result of its contraction. *l_i_* and *v_i_* are the length and contraction velocity of muscle *i*. When these signals come directly from the arm dynamics simulation they are not delayed. *q_s_* is a quaternion specifying the orientation of the upper arm. θ_e_ is the angle of elbow flexion. *e* is the distance between the hand and the target. [$\dot{e}$]^+^ is the positive part of the derivative of *e*. *l_i_*, *v_i_*, *q_s_*, ${\dot{q}}_{s}$, θ_*e*_, ${\dot{\theta }}_{e}$ are subject to a proprioceptive delay of 25 ms, whereas *e* and $\dot{e}$ are subject to a visual delay of 150 ms. The blocks inside the red and green dashed lines are used for the 4 models in the paper. The elements inside the red dashed square comprise the central controller in **Figures 5**, **7**, **9**, **11**. The blocks surrounded by the green dashed lines constitute the muscle, environment, and parietal cortex blocks in **Figures 5**, **7**, **9**, **11**. Implementation of the blocks is described in the Section 2.

The way our controller guides reaching starts by mapping the Cartesian coordinates of a target into the muscle lengths that the arm would have with the hand located at those coordinates. In order to make this mapping one-to-one we assume that the upper arm performs no rotation. The difference between the current muscle length and the target muscle length will produce a muscle stimulation, modulated by the contraction velocity (details in next subsection). The blocks labeled “inverse kinematics” and “feedback controller” in Figure 3 represent the computations of the central controller being described.

#### 2.2.1. Equations for the central controller

The central controller performs two tasks in order to reach for a target. The first task is, given the coordinates of the target, to produce the muscle lengths that would result from the hand being at those coordinates. The second task is to contract the muscles so that those target lengths are reached.

The first task (inverse kinematics) requires to map 3D desired hand coordinates into an arm configuration. The spatial configuration of the arm that leads to hand location is specified by 3 Euler angles α, β, γ at the shoulder joint, and the elbow angle δ. Our shoulder Euler angles correspond to intrinsic ZXZ rotations. In order to create a bijective relation between the 3D hand coordinates and the four arm angles we set γ = 0.

For a given target hand position we calculate the angles α, β, γ, δ corresponding to it. Using these angles we calculate the coordinates of the muscle insertion points, from which their lengths can be readily produced. When the muscle wraps around a bending line we first calculate the point of intersection between the muscle and the bending line. The muscle length in this case comes from the sum of the distances between the muscle insertion points and the point of intersection with the bending line.

The formulas used to calculate the angles α, β, γ, δ given hand coordinates (x,y,z) and the shoulder at the origin are:

(1) $$\alpha =\sin^{-1}\left(\frac{-x}{\sqrt{x^{2}+y^{2}}}\right),$$

(2) $$\begin{array}{l} \beta =\cos^{-1}\left(\frac{-z}{\sqrt{x^{2}+y^{2}+z^{2}}}\right)\\ \;-\cos^{-1}\left(\frac{x^{2}+y^{2}+z^{2}+L_{arm}^{2}-L_{farm}^{2}}{2(x^{2}+y^{2}+z^{2})L_{arm}}\right), \end{array}$$

(3) γ=0,

(4) $$\delta =\pi -\cos^{-1}\left(\frac{L_{arm}^{2}+L_{farm}^{2}-(x^{2}+y^{2}+z^{2})}{2L_{arm}L_{farm}}\right).$$

Where *L_arm_* and *L_farm_* are the lengths of the upper arm and forearm respectively. If we have the coordinates of a humerus muscle insertion point (as a column vector) at the resting position, then we can find the coordinates of that insertion point at the position specified by α, β, γ using the following rotation matrix:

(5) $$R=\left[\begin{array}{ccc} \begin{array}{c} \text{c}(\alpha )\text{c}(\gamma )-\text{s}(\alpha )\\ \text{c}(\beta )\text{s}(\gamma ) \end{array} & \begin{array}{c} -\text{c}(\alpha )\text{s}(\gamma )-\text{s}(\alpha )\\ \text{c}(\beta )\text{c}(\gamma ) \end{array} & \text{s}(\alpha )\text{s}(\beta )\\ \begin{array}{c} \text{s}(\alpha )\text{c}(\gamma )+\text{c}(\alpha )\\ \text{c}(\beta )\text{s}(\gamma ) \end{array} & \begin{array}{c} -\text{s}(\alpha )\text{s}(\gamma )+\text{c}(\alpha )\\ \text{c}(\beta )\text{c}(\gamma ) \end{array} & -\text{c}(\alpha )\text{s}(\beta )\\ \text{s}(\beta )\text{s}(\gamma ) & \text{s}(\beta )\text{c}(\gamma ) & \text{c}(\beta ) \end{array}\right]$$

where *c*(·) = cos(·), s(·) = sin(·).

The coordinates of insertion points on the forearm at the pose determined by α, β, γ, δ are obtained by first performing the elbow (δ) rotation of the coordinates in the resting position, and then performing the shoulder rotation (α, β, γ). Muscle lengths come from the distance between their insertion points, or between their insertion points and their intersection with the bending line. Details on how to determine whether a muscle intersects a bending line can be found in the function piece5.m, included with the source code. This function also obtains the point of intersection, which is the point along the bending line that minimizes the muscle length.

Once we have found target equilibrium lengths for the muscles, we must contract them until they adopt those lengths. To control the muscles we use a simple serial cascade control scheme. The length error *e_l_* of a muscle is the difference between its current length *l* and its equilibrium length λ. The velocity error *e_v_* is the difference between the current contraction velocity *v* (negative when the muscle contracts), and the length error *e_l_*:

(6) $$e_{l}=g_{l}(l-\lambda ),\;e_{v}=g_{v}(v+e_{l}).$$

The constants *g_l_*, *g_v_* are gain factors. For all simulations *g_l_* = 2, *g_v_* = 1. The input to the muscles is the positive part of the velocity error. This creates a force that tends to contract the muscle whenever its length exceeds the equilibrium length, but this force is reduced according to the contraction speed. At steady state the muscle lengths may or may not match the equilibrium lengths, depending on the forces acting on the arm. To promote stability the output of the central controller went through a low-pass filter before being applied to the muscles. Also, to avoid being stuck in equilibria away from the target, a small integral component was added, proportional to the time integral of the central controller's output.

### 2.3. Cerebellar model

The cerebellar model provides motor commands whenever an “error-prone area” of state space is entered. Each error-prone area consists of a point in state space (its center, or feature vector), and a kernel radius. To each error-prone area there also corresponds a “correction vector,” specifying which muscles are activated and which are inhibited when the error-prone area is entered. At each iteration of the simulation the distance between the currently perceived point in state space and the center of each error-prone area is obtained, and each correction vector will be applied depending on this distance, modulated by its kernel radius. The kernels used can be exponential or piecewise linear. The action of the cerebellar model is represented in Figure 3 by the block labelled “stored corrections.”

Learning in the model requires an error signal, which could be visual (such as the one that may be generated in posterior parietal cortex Desmurget et al., 1999), or could arise from muscle afferents. Block diagrams corresponding to the model with the visual and muscle error signals are in **Figures 5**, **7**, **9**, **11**. The visual error signal arrives with a delay of 150 ms. Each time the error increases its magnitude (its derivative becomes positive) this increases the probability of complex spikes; for each IO cell, this probability also depends on the current phase of its subthreshold oscillation (see next subsection). Complex spikes generate a new error-prone area. The feature vector associated with this area is the state of the system a short time span before the error increased; usually this time span will be half the time it takes for the error derivative to go back to zero, plus an amount of time comparable to the perceptual delay. For as long as the error derivative is positive, at each iteration we will record the efferent signals produced by the central controller, and when the derivative stops being positive we will obtain the average of all the recorded efferent signals. The correction is obtained from this average. The muscles are driven by the velocity errors, so these are the efferent signals collected during correction period. All the kernel radii were equal, so they have no change associated with learning.

Notice that if the error derivative remains positive, more complex spikes will be generated as different olivary nucleus cells reach the peak of their subthreshold oscillations. Thus, we have two gain mechanisms for a correction: one comes from the magnitude of the error derivative, which will promote a large response (and synchronous activity) of complex spikes; the second comes from the amount of time that the error derivative remains positive, since more inferior olivary nucleus cells reaching the peak of their subthreshold oscillations while this derivative is positive will mean a larger number of complex spikes, creating error-prone areas along the trajectory of the arm. Performance-wise, it is beneficial to have a sequence of error-prone areas rather than a single one, since the appropriate correction to apply will change as the arm moves.

When the new feature vector is too close to a previously stored one, or when we have already stored too many feature vectors, then the new feature vector will become “fused” with the stored feature vector closest to it. When two areas fuse they are both replaced by a new area whose feature vector is somewhere along the line joining the feature vectors of its parent areas, and likewise for its correction vectors.

#### 2.3.1. Algorithm for the cerebellum simulations

We will describe the part of the computational model that deals with the functions of a microcomplex (the file CBloop11c.m of the source code). To simplify the exposition, we do not consider the case when the maximum number of “feature vectors” have been already stored.

The input to the microcomplex model has components that represent error, and afferent/efferent signals. The error component consists of the distance between the hand and the target (the visual error), and its derivative (from which complex spikes are generated). The afferent information includes a quaternion describing the shoulder joint position, the derivative of this quaternion, an angle describing the elbow position, and this angle's derivative. The efferent input is the muscle input described in Section 2.2.1 (consisting of 11 velocity errors), and in addition, the desired shoulder position (expressed as a quaternion), and the desired elbow angle. The error and its derivative arrive with a visual delay of 150 ms. The rest of the information arrives with a proprioceptive delay of 25 ms.

The output of the microcomplex consists of 11 additional signals that will be added to the muscle inputs.

The algorithm's pseudocode is presented next. An unhandled spike is a complex spike whose “context,” consisting of the afferent/efferent signals and the error briefly before the spike, has not been stored as a “feature vector.” A “feature vector” is a context associated with a motor correction.

At each step of the simulation:

**1:** Generate complex spikes using the error derivative

**2:**

**if** there are unhandled spikes **then**

   **if** If the error derivative is no longer positive, and the time since the spike doesn't exceed 250 ms **then**

      **2.1.1:** Store the context corresponding to the unhandled spike as a new feature vector

      **2.1.2:** Store the motor correction associated with the new feature vector

   **end if**

**end if**

**3:** For each feature vector, calculate its distance to the current context, and add its motor correction to the output as a function of that distance

In step 2.1.1, the stored feature vector consists of the context as it was $\tau_{v}-\tau_{p}+\frac{t-t_{cs}}{2}$ milliseconds before the complex spike, with τ*_v_* being the visual delay, τ*_p_* the proprioceptive delay, *t* the current time, and *t_cs_* the time when the complex spike arrived.

In step 2.1.2, the motor correction that gets stored is the average motor input from $\left(t_{cs}-\tau_{v}+\tau_{p}\right)$ to $\left(t-\tau_{v}+\tau_{p}\right)$.

The output that the microcomplex provides at each simulation step is obtained using radial basis functions. The distance between the current context and each feature vector is calculated, and those distances are normalized. The contribution of each feature vector to the output is its corrective motor action scaled by an exponential kernel using that normalized distance. Let *f*(*i*) be the *i*-th feature vector, and *w*(*i*) its associated correction. Let *v* denote the vector with the current context information. We first obtain a distance vector *D*, whose components are: *D*(*i*) = ||*f*(*i*)−*v*||^2^.

The distance vector is normalized as $D_{N}=(\sqrt{M_{F}}/\|D\|)D,$ where *M_F_* is the maximum number of feature vectors allowed. The contribution of feature *i* to the output is *F*(*i*) = *w*(*i*)*e*^γ*D_N_*(*i*)^, with γ specifying the kernel radius.

### 2.4. Inferior olivary module

The process of generating complex spikes when using the visual error is explained next. By “complex spike” we mean a signal indicating that a correction should be stored. There are *N* inferior olivary nucleus cells, from which *N*_3_ are assumed to oscillate at 3 Hz, and *N*_7_ are assumed to oscillate at 7 Hz. The phases of both cell subpopulations are uniformly distributed so as to occupy the whole range [0, 2 π] in the equation below. Let ϕ(*i*) denote the phase of cell *i*, and α(*i*) denote its angular frequency. The probability to spike at time *t* for cell *i* is calculated as:

(7) $$P_{CS}^{i}(t)=p\frac{\cos[\alpha (i)(t-\phi (i))]+1}{\left(1+e^{\left(5\;-\;E\right)})(1+e^{\left(30\;-\;15{\left[E^{\prime }\right]}^{+}\right)}\right)}$$

Where *p* is a constant parameter, *E* is the visual error, and [*E*′]^+^ is the positive part of its derivative. At each step of the simulation a random number between 0 and 1 is generated for each cell. If that number is smaller than *P^i^_CS_*, and the cell *i* has not spiked in the last 200 ms, then a complex spike is generated.

Complex spikes are less likely to be generated when the error is small. When the hand is close to the target it is likely that it oscillates around it. Generating cerebellar corrections in this situation could be counterproductive, as the angle between the hand and the target changes rapidly, and so do the required corrections. In our idealized cerebellum (see Supplemenatry Material) there are conditions ensuring that no corrections are created when the angle between the hand and the target has changed too much. Since there is no obvious biological way to measure the angle between the hand and the target, we just avoid generating corrections when the hand is close to the target. Another mechanism present in our computational simulations to deal with this problem is that no corrections are stored if the time between the complex spike and the time when the error stops increasing is more than 250 ms.

Generating complex spikes when using the proprioceptive error follows a simpler procedure. For each muscle three conditions must be satisfied for a “complex spike” to be generated: (1) its length *l* is increasing, (2) *l* is longer than it's target value λ, and (3) no complex spikes have been generated for that muscle in the last 200 ms. A variation described in the Section 3 adds a fourth condition: (4) the visual error must be increasing (*E*′ > 0).

### 2.5. Generating corrective muscle activity

In this paper there are three different methods to determine the corrective motor commands that become associated with points of state space where the error increases.

**Figure 4:**
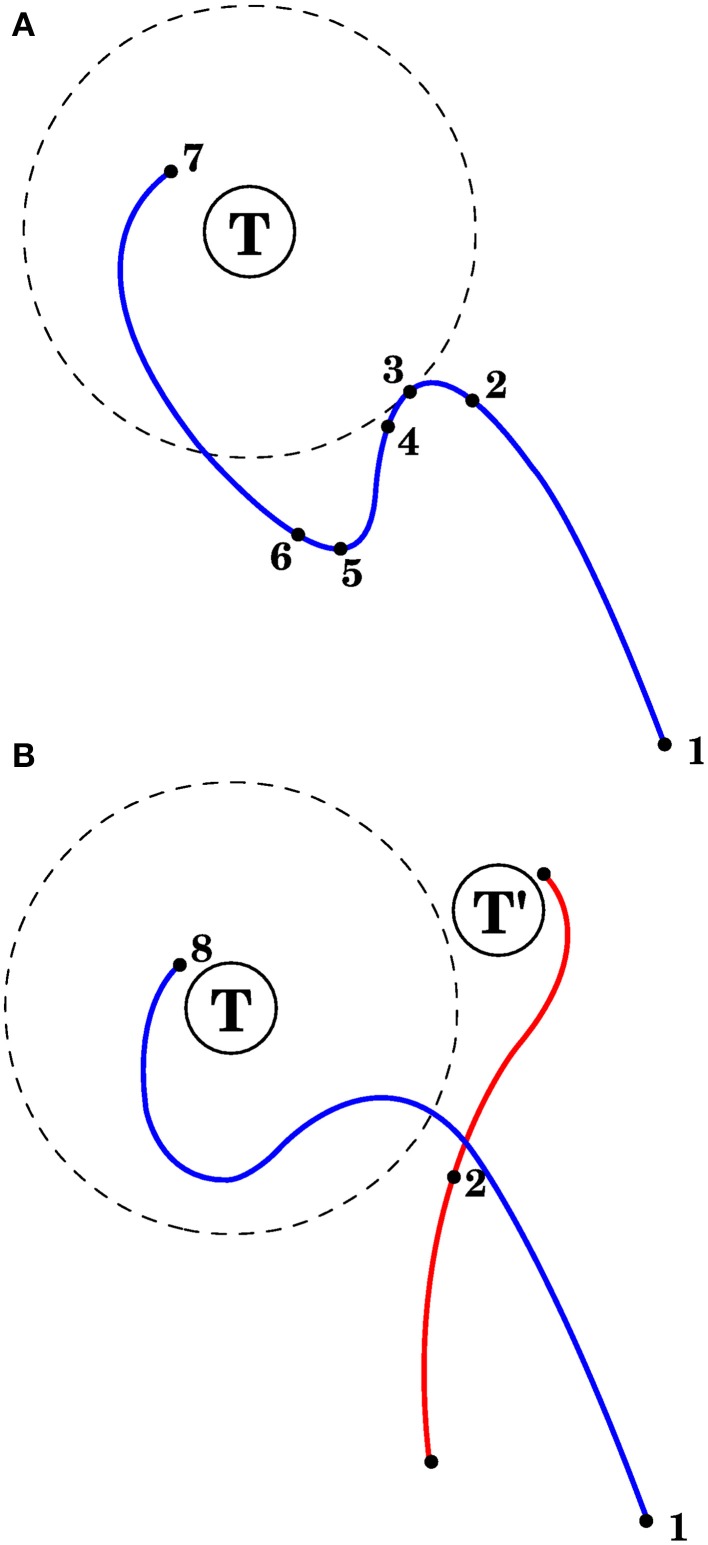
**Correcting reaching errors. (A)** Schematic trajectory of the hand as it reaches for target T in 2 dimensions. Seven points of the trajectory are illustrated, corresponding to seven important points in time with different afferent/efferent contexts. **1**. Initial position of the hand. **2**. The context at this point will be associated with the correction. **3**. The error begins to increase. **4**. Complex spikes reach the cerebellar cortex in response to the error increase. **5**. The error is no longer increasing. **6**. The context at point 2 becomes associated with a correction, which could consist of the mean efferent activity (roughly) between points 3 and 5. **7**. Final hand position. **(B)** After the correction in **(A)** is learned, and the same reach is attempted, the trajectory will be modified upon approaching point 2, with the correction being applied anticipatively (blue line). Notice that a different trajectory (red line) that passes through the spatial location of point 2 may not elicit the correction learned in **(A)**. This is because the correction is applied when its associated context is near to the current context (which is a point in state space); those contexts contain velocities, efferent activity, and target location in addition to the arm's spatial configuration.

The first method, in model 1 of the Section 3, is used with visual errors. The corrective commmand consists of the average efferent commands produced from the point when the error started to increase until the error stopped increasing (points 3 and 5 in Figure 4A).

The second method, in models 2 and 3, is used with proprioceptive errors. If a complex spike is generated for a muscle, the corrective command is simply a slight contraction of that same muscle.

The third method is used with visual errors, and is applied in model 4. The corrective command for muscle *i* will be proportional to the product *c_i_* = [< *l_i_* > −λ_*i*_]^+^[$\dot{l}$_*i*_]^+^, where *l_i_* is the length of muscle *i*, < *l*_*i*_ > is the average of that length through a brief period before the error stopped increasing (e.g., a brief period between points 3 and 5 in Figure 4A, λ*_i_* is the target length for muscle *i*, $\dot{l}$_*i*_ is the derivative of the length, and [·]^+^ returns the positive part (and zero otherwise).

## 3. Results

### 3.1. Implementing the architecture in a reaching task

We hypothesize that the role of the cerebellum in motor control is to associate afferent and efferent contexts with movement corrections; in the case of reaching the controller involves the cortex, basal ganglia, brainstem, and spinal cord. The role of the central controller is to reduce error, and we begin by assuming that the role of the cerebellum is to anticipatively apply the corrections of the central controller. (model 1 below). How this could happen for the case of reaching is described in Figure 4. Before an incorrect motion is made (moving the hand away from the target), the mossy fibers reaching the granule layer have afferent and efferent information that could predict when this error will occur. When the error does increase during a reach, this is indicated by complex spikes, while the central motor controller is acting to correct the error. The cerebellum associates the afferent and efferent information of granule cells shortly before the increase in error with the motor actions required to correct it, using climbing fiber activity as the training signal. The corrective motor actions can be those that the central motor controller produces in order to stop the error from increasing, which come shortly after the onset of error increase; thus the cerebellum doesn't have to obtain those actions itself, it can merely remember what the central controller did. This idea is related to Fujita's feed-forward associative learning model (Fujita, 2005). Other ways to obtain the corrective motor actions are described in the models below.

As mentioned in the Introduction, we created mathematical and computational models implementing these ideas. The mathematical model and the results of its analysis are described in the Discussion. The full mathematical treatment is in the Supplementary Material. The elements of the computational models are described in the Section 2. In the remainder of the Results we present the outcome of simulations using four computational models with basic variations of our cerebellar architecture. All these computational models use the same central controller and the same arm and muscle models.

The physical simulation of the arm used for this study used no friction at the joints. The muscles had limited viscoelastic properties and the control signals had low gain. Under these conditions, the arm under the action of the central controller alone tended to place its distal end at the target slowly (in around 1.5 s) and with some oscillations, even in the absence of gravity forces. Introducing a 25 ms proprioceptive delay resulted in larger oscillations, and the hand no longer reached the target with arbitrary accuracy, but would instead oscillate around it in a non periodic fashion. Moreover, certain positions of the target would cause the arm to become unstable, leading to chaotic flailing.

To test that the cerebellar corrections could gradually reduce the error as learning progressed through successive reaches, we selected 8 target locations and simulated 8 successive reaches to each target. From these 8 targets one of them (target 2) produced instability of the arm when no cerebellar corrections were applied. The same 8 targets were used for the four models presented here. Figure 2 presents a visualization of the arm's geometry, and of the 8 targets.

### 3.2. Simulation results

#### 3.2.1. Model 1: visual errors, efferent copies to generate corrections

**Figure 5:**
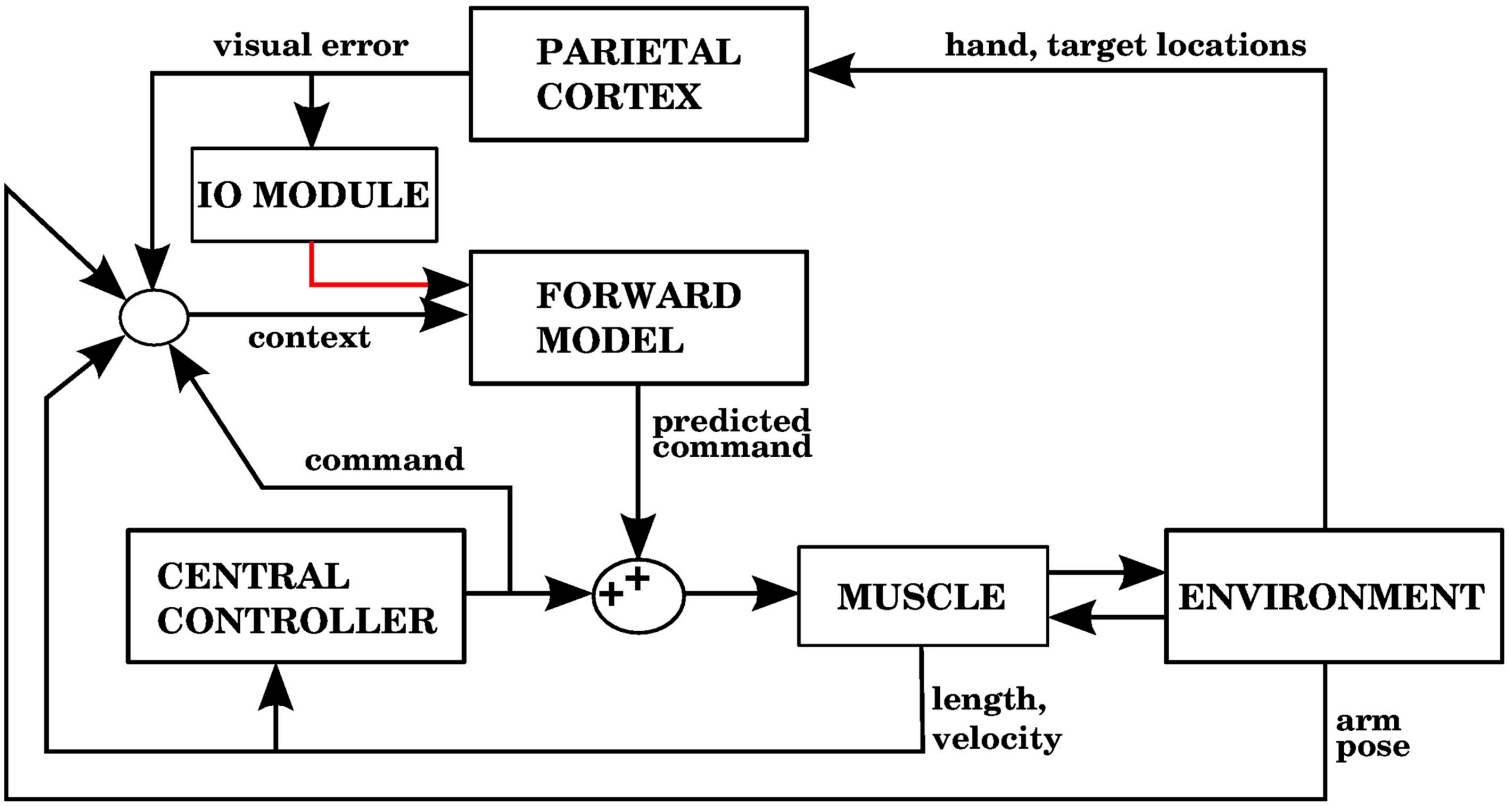
**Computational model with the visual error signal, and a corrective command that is obtained from the efferent commands produced by the central controller (model 1 in the text)**. This is the same model depicted in Figure 3, but at a slightly higher level of description. The error (assumed here to be obtained in parietal cortex) consists of the distance between the hand and the target, and increases of this error cause the forward model to associate the context with a correction. The learning signal, produced when the error increases, is denoted by the red line. The forward model corresponds to the stored corrections in Figure 3, and the environment corresponds to the arm dynamics simulation.

We first considered the case when complex spikes were generated when the distance between the hand and the target increased, according to Equation (7). The corrective muscle commands were proportional to the average of the efferent commands produced between the onset of error increase and the time when the error no longer increased (the period between points 3 and 5 in Figure 4). Figure 5 presents a block diagram indicating the signals and modules involved in this model.

**Figure 6:**
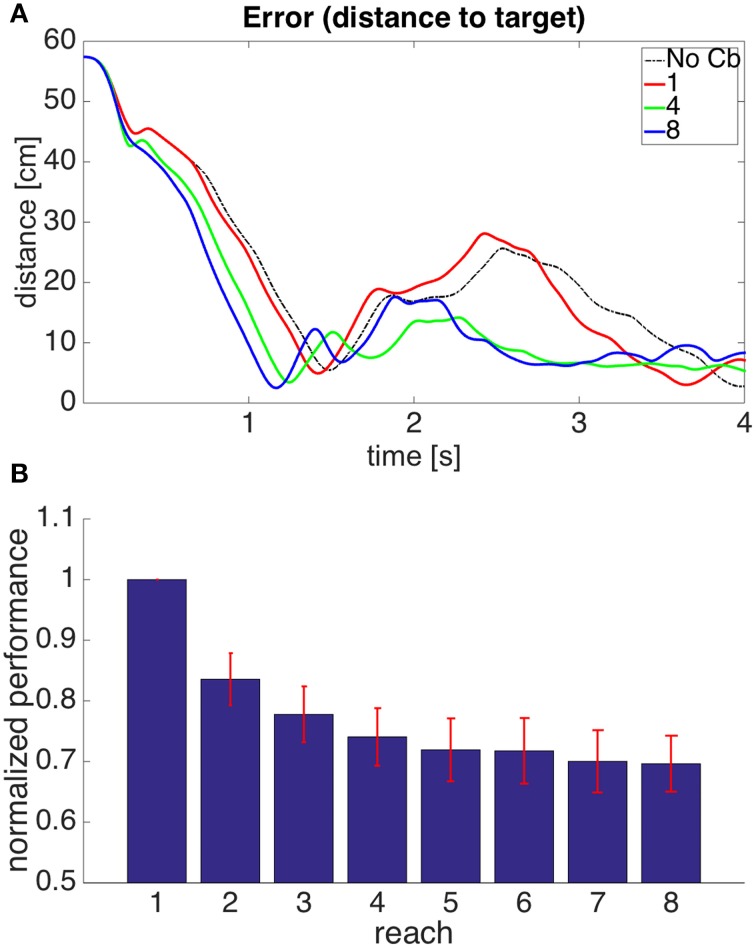
**Results for Model 1. (A)** Distance between hand and target through 4 s of simulation time for the first, fourth, and eighth reaches to target 7. The cerebellar system was trained using the distance between the hand and the target as the error, and the target had coordinates X = 20 cm, Y = 40 cm, Z = −20 cm. The dashed line, labeled “No Cb,” shows the error when the arm was actuated by the central controller exclusively. Notice how the first reach (red line) is slower, and oscillates away from the target after approaching it. This is significantly improved on the eighth reach (blue line). **(B)** Integral of the distance between the hand and the target during the 4 s of simulation for the 8 successive reaches. Each bar corresponds to the value obtained from averaging this performance measure across the 8 targets. The bars were normalized by dividing between the value for the first reach. For each bar its standard error measure (*S.D*./$\sqrt{8}$) is shown using the red lines at its upper edge.

Figure 6A shows the evolution through time of the distance between the hand and the target in the 1st, 4th, and 8th reaches toward a representative target. To measure the success of a reach we obtained the time integral of the distance between hand and target through the 4 s of simulation for each reach. Smaller values of this performance measure indicate a faster, more accurate reach. Figure 6B shows our performance measure for each of the 8 successive reaches, averaged over the 8 targets.

Figure 6 shows that on average the performance increases through successive reaches. The error may not decrease monotonically, however, since the correction learned in the last trial may put the system in a new region of state space where new errors can arise within the time of the simulation. Eventually, however, the hand comes close to monotonically approaching the target. The instability present in the second target dissappeared on the second reach (not shown).

Although this model improves the performance of the reach, it can't be considered biologically plausible unless we understand how the outputs at the deep cerebellear nucleus could become associated with the corrections they presumably apply. Basically, the problem is that if all microcomplexes receive the same learning signal (increase in visual error), then all the DCN populations will learn the same response, and the arm would express all possible corrections upon entering an error-prone area of state space. In the Discussion we elaborate on this. In the rest of the Section 3 we present 3 alternative models were the corrections to be applied are not learned from efferent copies of the commands to the arm, but from proprioceptive signals.

#### 3.2.2. Model 2: proprioceptive errors, individual muscle corrective signals

**Figure 7:**
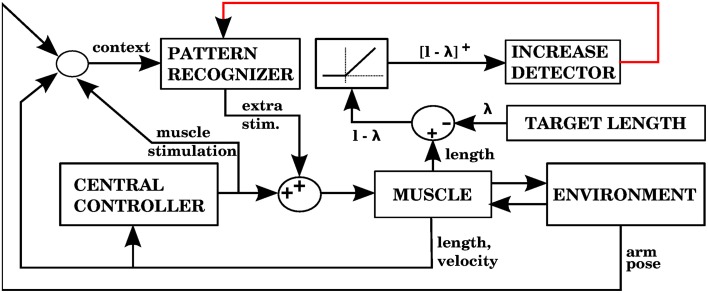
**Model with the proprioceptive error signal, and a corrective command that is simply a contraction of the muscle that produced the error signal (model 2 in the text)**. The error is the muscle length *l* minus the target length λ. This target length comes from the central controller. When *l* − λ is positive, increases of this error in a particular context will cause the pattern recognizer to apply an anticipative contraction when that context arises. The pattern recognizer corresponds to the block of stored corrections in Figure 3, and the increase detector corresponds to the IO module.

Using the equilibrium point hypothesis in the central controller has the distinct advantage that we know the lengths at which the muscle stops contracting (called target lengths in this paper). A simple way to detect errors could be to monitor when a muscle is longer than its target length, but is nevertheless elongating. A simple way to correct that error is to contract that muscle a bit more. The multidimensional task of applying corrections during 3D reaching is thus reduced to a group of one dimensional tasks corresponding to individual muscle groups. Figure 7 shows a block diagram implementing these ideas as done in our second model.

**Figure 8:**
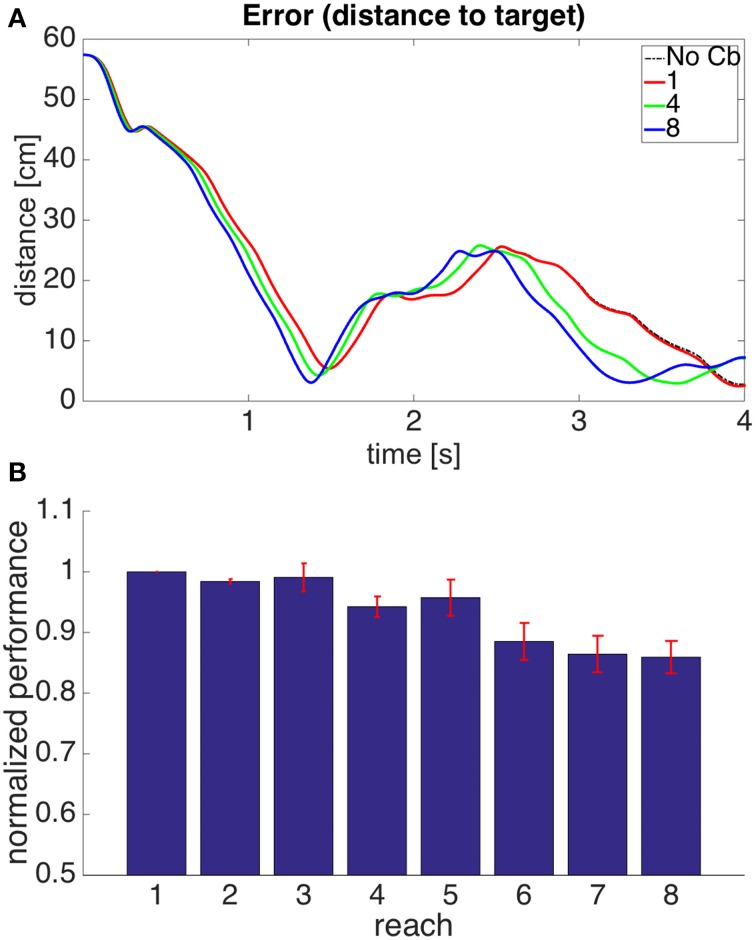
**Results for Model 2**. The cerebellar system was trained using an error signal produced when muscles became larger than their target value. **(A,B)** Refer to Figure 6 for interpretation.

Figure 8 shows the results of using a model where the errors are detected and corrected at the level of individual composite muscles, as just described. It can be observed that improvement is slower than in the case of the previous model. For example, the instability of the second target only dissappeared during the sixth reach (not shown). In our simulations of model 2 the cerebellar corrections could lead to instability unless we use small kernel radii and small amplitude for the corrections. A possible reason for this is that our central controller does not specify an optimal temporal sequence of muscle contractions, but instead specifies a static set of target lengths. The trajectory of muscle lengths that leads the hand in a straight line toward the target may not have those lengths monotonically approaching the target lengths. On the other hand, our system generates an error signal whenever that approach is non monotonic. This inconsistency is the price of using one-dimensional signals to approach an error that arises from the nonlinear interaction of several independent variables. The next model uses a simple approach to try to overcome this problem.

#### 3.2.3. Model 3: proprioceptive errors with visual error constraint, individual muscle corrective signals

**Figure 9:**
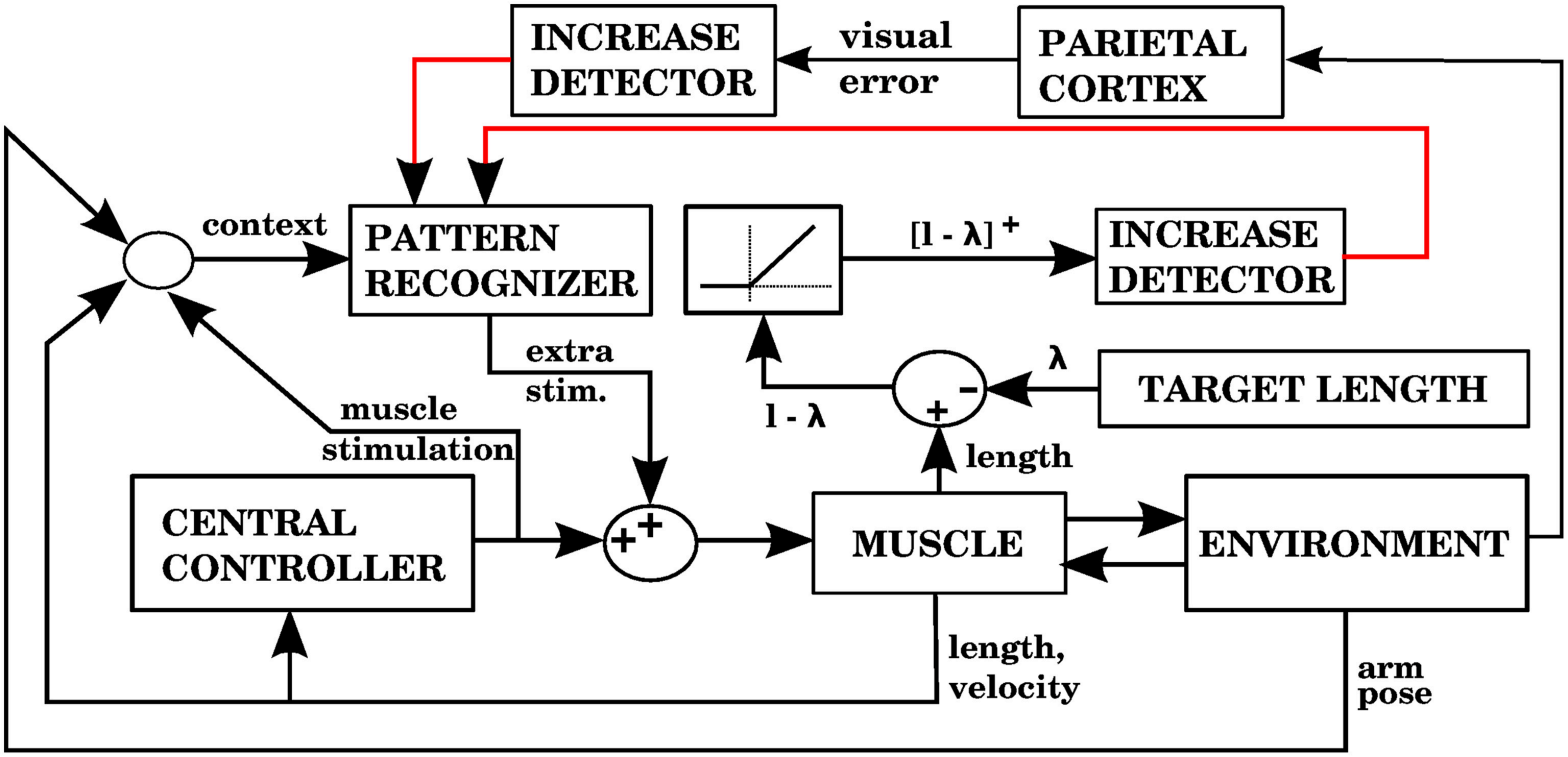
**Model with the proprioceptive error signal, a visual error constraint, and a corrective command that is simply a contraction of the muscle that produced the error signal (model 3 in the text)**. Notice that this is similar to the model in Figure 7, but we have an additional learning signal entering the pattern recognizer. This additional signal ensures that corrections are stored only when the visual error is increasing.

In the previous model the gain of the corrections and their area of application in state space had to remain small because there can be some inconsistency between the error signals from individual muscles and the visual error. A muscle continuing to elongate past its target value does not imply that contracting it will bring the hand closer to the target. A simple way to address this is to add the necessary condition that if a correction is to be stored, the visual error should be increasing. Corrective signals will thus arise when the muscle is elongating beyond its target length, and the hand is getting away from the target. In this way, even if the muscle lengths are getting away from their target values, no corrections will be stored when the hand is approaching the target monotonically. Figure 9 shows how the architecture of model 2 is augmented with visual errors in order to produce model 3.

**Figure 10:**
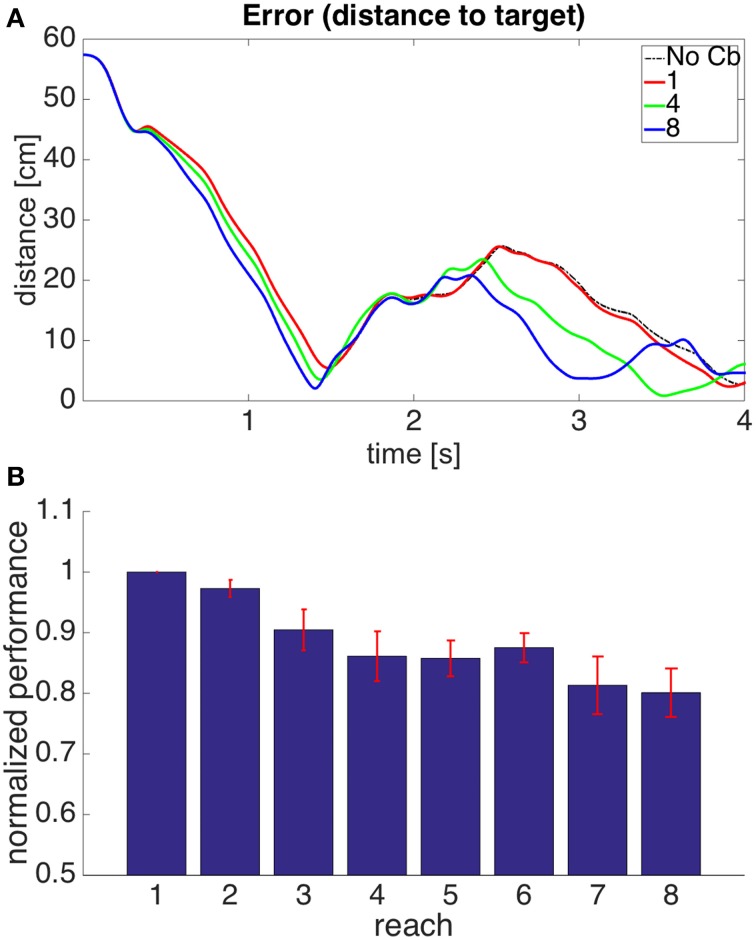
**Results for Model 3**. The cerebellar system was trained using an error signal produced when muscles became larger than their target value, with the additional constraint that the error (distance between hand and target) had to be increasing. **(A,B)** Refer to Figure 6 for interpretation.

Figure 10 shows the results of using a such a model. Using the additional constraint permits larger gains in the corrections and larger kernel radii than those used in model 2. This is reflected by a larger increase in performance. This increase, however, is still not as good as that seen in model 1. The visual error is what we really want to reduce, and there is a limit to how much this can be done when the error signals are triggered at the level of muscles, as the visual error and the proprioceptive error are not entirely equivalent. This is addressed by the next model.

#### 3.2.4. Model 4: visual errors, proprioceptive corrective signals

As discussed above, visual errors are the most appropriate to improve performance, so in this model we use them, just as in model 1. Unlike model 1, we don't use the commands from the central controller in order to create the corrections. We must then find a way to solve the distal error problem without the central controller. A way to do this is to create corrections similar to the signals that indicated error increase in models 2 and 3.

**Figure 11:**
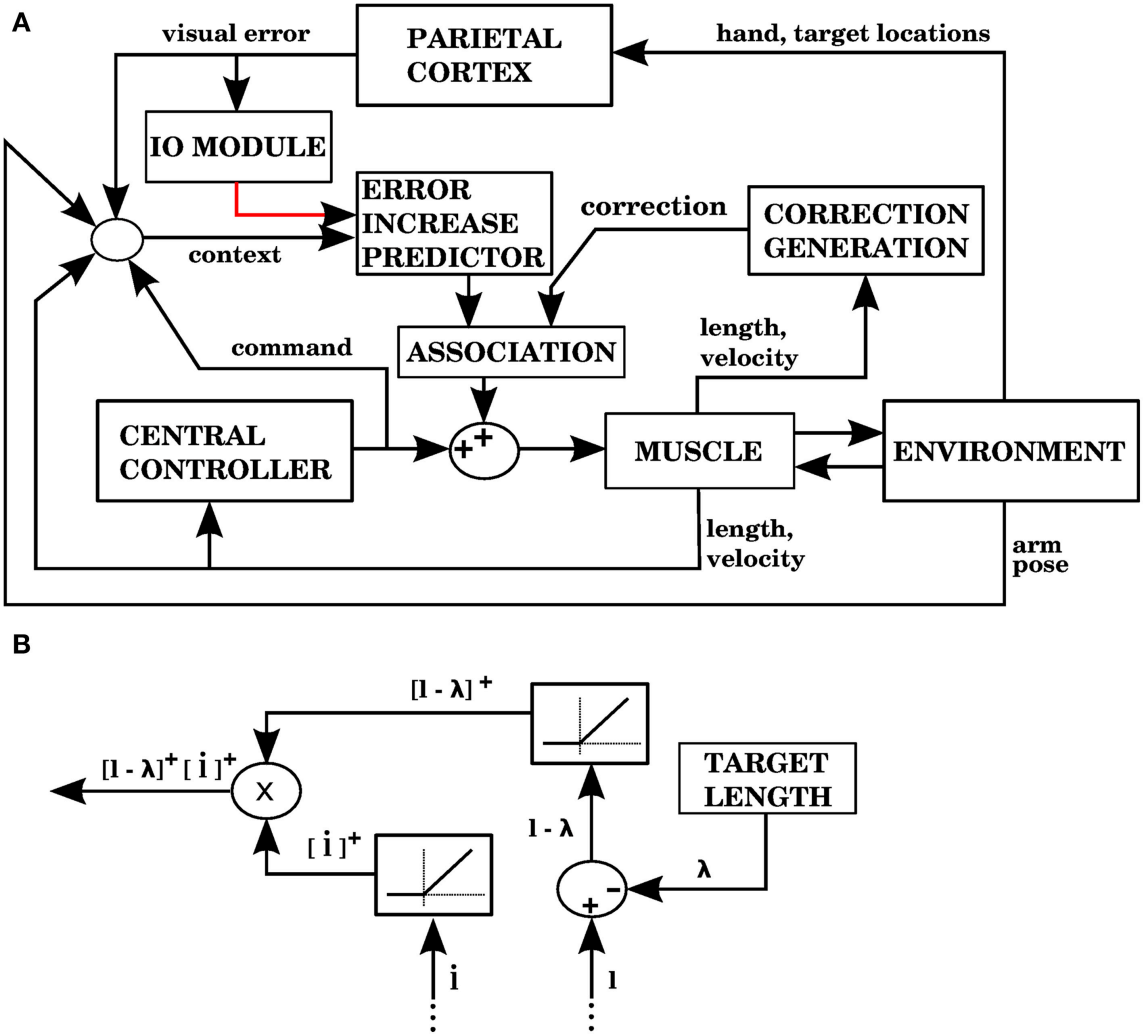
**Model with visual errors and proprioceptive error signals (model 4 in the text). (A)** The visual error signal used by this model is the same one as in model 1, but unlike model 1, the correction associated with an error is not a copy of a command from the central controller. In this case, the correction is generated from proprioceptive information (muscle length and contraction velocity) in the block labeled as “CORRECTION GENERATION” (expanded in **B**). This correction is to be applied when the error is predicted to increase. In the block labeled “ASSOCIATION” a signal predicting the onset of error increase becomes associated with the correction, so that when the increase in error is predicted the correction is applied. **(B)** The computations performed in the “CORRECTION GENERATION” block of **(A)**. For each muscle, its length *l* and contraction velocity $\dot{l}$ are received, along with a target length λ. The correction consists of the product between the positive parts of *l* − λ and $\dot{l}$.

Model 4 generates error signals (complex spikes) when the hand is getting away from the target according to Equation (7), just like model 1. Figure 11A shows the signals and modules implied by model 4. For each muscle, the correction associated with an error signal is proportional to two factors: how much longer the muscle is than its target value, and how fast its length is increasing (Figure 11B). The block that associates contexts with predicted increases in error (labeled “ERROR INCREASE PREDICTOR”) is identified with the cerebellum, while the “CORRECTION GENERATION” module is identified with muscle afferents and spinal cord neurons. We assume that the predictions of error increase from the cerebellum become associated with the corrections generated at the level of the spinal cord. This is elaborated in the Discussion.

**Figure 12:**
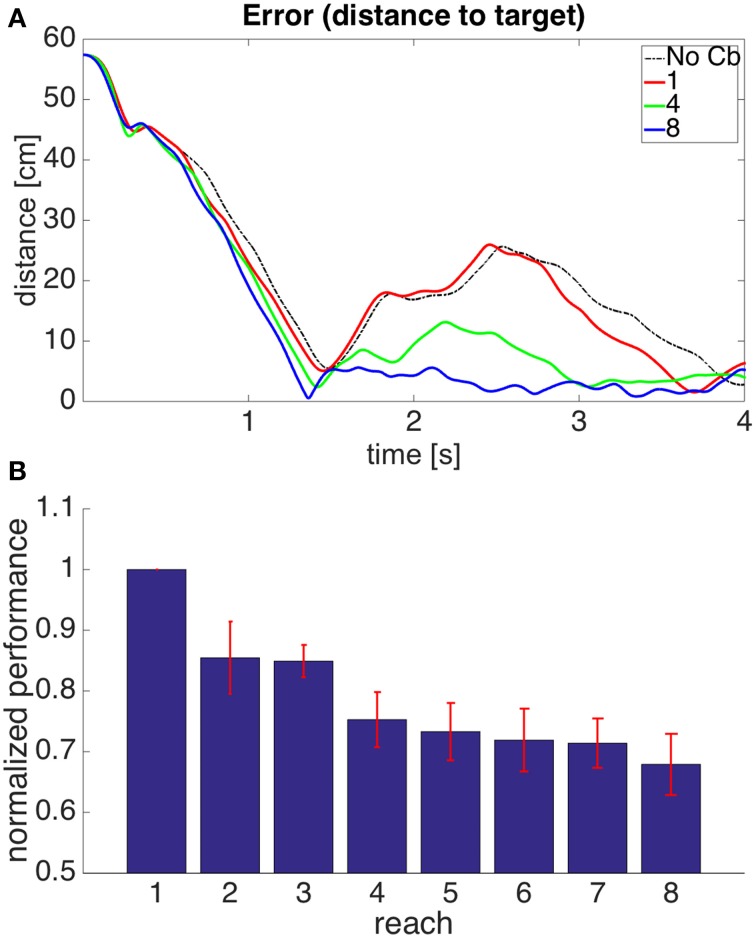
**Results for Model 4**. The cerebellar system was trained using the same error signal as in model 1 (Figure 6), but the corrective commands were produced from muscle proprioceptive signals. **(A,B)** Refer to Figure 6 for interpretation.

Figure 12 shows the performance of model 4. It can be seen that the error reduction is comparable to that of model 1, but using a novel solution to the motor error problem based on the assumption that the muscle is controlled through an equilibrium length.

## 4. Discussion

As research on the cerebellum continues, it becomes increasingly clear that although cerebellar microzones have a uniform architecture, the role they play in various systems can be different depending on their input and output connections. For example, cerebellar microzones could implement either forward or inverse models (Popa et al., 2012; Ito, 2013; Porrill et al., 2013). Cerebellar architectures such as feedback-error learning (Kawato and Gomi, 1992, Figure 1B) and the recurrent architecture (Porrill and Dean, 2007, Figure 1A) specify the connectivity of cerebellar microzones, and the computational role they would therefore play.

We have presented an architecture in which the cerebellum reduces errors associated with climbing fiber activity when that activity arises from the increase in some error measure. Instead of assuming that complex spikes encode the magnitude of some performance error, we have assumed that they are generated when the derivative of the error becomes positive. This leads to a sparse code that generates a forward model for anticipative corrections. This forward model exists only in locations of state space where the error is prone to increase, and predicts a corrective command, not the output of the controlled object. Very importantly, the identity of the error signal does not need to imply the dimension along which the correction should be made. Although we have assumed that the central controller uses closed-loop feedback, this is not necessary for our first model. Our architecture has the potential to explain the presence of predictive and feedback performance errors in Purkinje cell simple spikes (Popa et al., 2012, 2013), the correlation of complex spikes with both sensory and motor events (Ito, 2013), the sparsity of complex spikes, and as discussed below, the role of the cerebellum in nonmotor operations (Ito, 2008; Buckner, 2013; Koziol et al., 2014; Popa et al., 2014). A possible way to discard our architecture in a particular system is when errors that are not increasing or changing elicit a sustained complex spike response. By “errors,” we refer not only to performance errors, but in general to signals that merit a behavioral response, such as an unexpected perturbation, or a potentially nociceptive stimulus.

We have explored our architecture in the context of reaching in 3D space. In addition to the mathematical treatment described below, we showed that the equilibrium point hypothesis gives our architecture the ability to solve the motor error problem in a novel way, using proprioceptive muscle signals (models 2,3, and 4). The success of model 4 suggests that we can predict errors using visual signals, and generate corrections using proprioceptive signals. It is clear that we can provide predictive control without the need to predict the kinematic or dynamic state variables of the controlled plant. Moreover, a signal which very loosely represented the positive part of the error derivative is sufficient to train our predictive controller. The type of corrections that our model cerebellum provides tend to avoid episodes where the hand gets away from the target; this is important when using a controller based on the lambda model of the equilibrium-point hypothesis (Feldman and Levin, 2009). A controller that only specifies a set of target muscle lengths (and not a trajectory of such lengths) may produce reaches by simultaneously contracting all the muscles whose lengths are longer than their desired lengths. This, in general, will not result in a straight-line reach. What the cerebellar controller does is to modify the activity of antagonist muscles at different points of the trajectory so that the hand monotonically approaches its target, producing a reach that is closer to a straight line.

All four models in this paper avoid or solve the redundancy problem. In Section 2.5 three ways of generating corrective motor commands were described. When the corrective output is generated from an efferent copy of the central controller (model 1), the redundancy problem is avoided, as it is assummed that this is handled by the central controller (the recurrent architecture avoids the redundancy problem in a similar manner). For the two other ways of generating corrective commands (in models 2,3,4), the redundancy problem is solved as soon as equilibrium lengths are given. Notice that equilibrium lengths determine the final position of the arm uniquely, as the viscoelastic properties of muscles lead the arm toward a configuration of minimal potential energy.

### 4.1. The mathematical model

In our mathematical model the hand is considered to be a point mass, and the central controller applies a force applied to this mass, always pointing to the origin, which is considered to be target. This constitutes a central force system, and as in the case of planetery motion under gravity forces it will tend to produce elliptical trajectories around the origin.

We modelled the “cerebellum” as a system that would apply impulsive forces to the point mass whenever particular regions of state space were entered, and proceeded to prove that such a cerebellum will continue to reduce the angular momentum in the trajectory until it either gets close enough to the target, or until it becomes circular. Circular trajectories do not ellicit cerebellar corrections because the error signal (distance between the hand and the target) does not increase. This is a shortcoming of generating learning signals only when the error increases.

The crucial part of this mathematical treatment is specifying when cerebellar corrections will be created, and for each cerebellar correction what will be the impulse vector associated with it. The cerebellar controller is characterized by three numbers: a speed threshold, a distance threshold, and a gain. A cerebellar correction is created whenever two conditions are met: the error begins increasing faster than the speed threshold, and it grows beyond the distance threshold.

The impulse associated with a correction is obtained by integrating the central controller's force, from the time when the error began to increase, until a stop time is reached; this is then multiplied by the gain. Specifying the integration stop time correctly is very important, and in our model we obtain it as the largest time when three conditions are all satisfied, namely: (1) the error is still increasing faster than the speed threshold, (2) the mass hasn't rotated around the origin more than π/2 radians, (3) the corrective impulse is not strong enough to reverse the radial velocity of the point mass. The first condition ensures that we only integrate forces that are contributing to stopping the error increase. The third condition exists so the corrective impulse is not strong enough to reverse the velocity of the mass, potentially bringing instability.

The second condition for the stop time ensures that the impulse vector roughly points in the opposite direction of the error's velocity vector. This condition is akin to the strictly positive real (SPR) condition of adaptive filter models (Porrill et al., 2013). The SPR condition states that the error signal used to train the filter should not have a phase shift of more than 90° at any frequency with respect to the actual error signal. In other words, the SPR condition states that the used error signal should be positively correlated to the error, whereas our second condition for the integration stop time states that the corrective signal should be negatively correlated with the increase in error. In the next subsection the subthreshold oscillation of inferior olivary nucleus cells is linked to the second condition for the integration stop time.

The mathematical treatment of our model points to several potential shortcomings implied in the three conditions for the integration stop time. These shortcomings are only strengthened by the fact that the arm does not exactly act as a central force on the hand. The ability of the cerebellar corrections to generalize properly to points in a ball surrounding an original correction point depends on how much the angle between the error's velocity and the corrective impulse change inside that ball. The arm exerting forces that don't point toward the target could reduce its negative correlation with the error velocity. This is a reason why the computational simulations in this paper (particularly model 1) are an important validation of our mathematical ideas.

### 4.2. The contents of climbing fiber activity

What the climbing fibers (CF) encode is still a contentious issue, and different assumptions lead to different models of cerebellar function. One set of assumptions is that the CF activity encodes performance errors involving the neuronal circuits of the PCs receiving those CFs. CF activity has indeed been found to be related to performance errors and unpredicted perturbations (Stone and Lisberger, 1986; Yanagihara and Udo, 1994; Bloedel and Bracha, 1998; Kitazawa et al., 1998), but it also has been found to correlate with both sensory and motor events, so that the nature of what is being encoded remains controversial (Bloedel and Bracha, 1998; Anastasio, 2001; Llinas, 2011).

We have assumed that complex spikes signal an increase in error, like the distance between the hand and a target, or the distance between the hand and its intended point in the trajectory. This is different from assuming that complex spikes perform a low-frequency encoding of the error (Kitazawa et al., 1998; Schweighofer et al., 2004; Kitazawa and Wolpert, 2005) because our onset signal doesn't track the error's magnitude, it is only related to the positive part of the error's derivative. Moreover, this climbing fiber signal does not require high firing rates, and the magnitude of the error correction could be obtained through several mechanisms such as cumulative learning through time, graded complex spikes (Najafi and Medina, 2013; Yang and Lisberger, 2014), or complex spike synchrony. In practice, for our models 1 and 4 we assume that the inferior olivary nucleus produces the output specified by Equation (7) of the Section 2. For models 2 and 3 the signal provided by the inferior olivary is similar in nature (an increase in error), but the error comes from muscle lenghts (model 2), or from a combination of muscle lengths and visual error (model 3). Details are in the Materials and Methods.

A noteworthy aspect of our computational simulations when using visual errors (models 1 and 4) is that we included an inferior olivary module that considered a number of units with subthreshold oscillations. This was done because such a module confers specific advantages in our architecture. Our second condition for the integration stop time in the mathematical model is more likely to be satisfied when the integration stop time is short. This means that instead of having a single large correction associated with an error prone area, it may be better to have several smaller corrections along the trajectory of the arm in state space during episodes of error increase. Our computational model of the inferior olivary module uses the subthreshold oscillations of IO cells as a mechanism to generate sequences of complex spikes during episodes of error increase, instead of having all IO cells firing simultaneously when there is an increase in error. The increase in error stimulates all IO cells targeting a microcomplex, but only those near the peak of their subthreshold oscillation will respond. As long as the error continues to increase, the IO cells nearing the peak of their oscillations will tend to activate.

To precisely convey the timing of increase onsets and to encourage stability it is important to have a wide range of phases in the subthreshold oscillations of inferior olivary cells (Jacobson et al., 2009), which largely depends on the coupling strength of olivary gap junctions (Long et al., 2002). The complex desynchronized spiking mode (Schweighofer et al., 1999) has a wide range of phases, as assumed in our simulations. We model the subthrehold oscillations of the IO cells so that the probability to spike for each cell depends on both the strength of the input signal and the phase of the subthreshold oscillation. Larger increases in the error produce stronger input signals to the inferior olivary, which are reflected by a larger number of neurons responding; thus, for any short time interval, the magnitude of the error increase is reflected by the number of inferior olivary cells spiking in synchrony. The inhibitory feedback from the cerebellar nuclear cells, in addition to functioning as a negative feedback system to control simple spike discharges (Bengtsson and Hesslow, 2006), could also help to avoid large clusters of synchronized inferior olivary cells, so as to maintain the complex desynchronized spiking mode.

### 4.3. From DCN activity to behavioral responses

If the group of Deep Cerebellar Nucleus (DCN) cells in one microcomplex stimulate only one muscle (or a set of agonists muscles), it is easy to see how in models 2 and 3 the right error signals for a given microcomplex come from the muscles affected by their DCN cells. In this case cerebellar modules can work as 1-dimensional systems, with an adaptive filter system as the one in Fujita (1982) or Chapeau-Blondeau and Chauvet (1991) being sufficient to perform the corrections. In our simulations, however, the increase in performance in models 2 and 3 was smaller than that in models 1 and 4, which used visual errors. This, we concluded, was the cost of using 1-dimensional errors to correct an error that is multidimensional (the distance between the hand and the target).

On the other hand, models 1 and 4 present a difficulty when considering why the activity of a given DCN cell activates the right muscles for a correction. As mentioned before, in models 1 and 4 there is only one learning signal (visual error increase), which would be the same for all microcomplexes. This implies that all microcomplexes would learn the same response, and entering an error-prone region of state space would elicit the responses associated with all DCN cells. Models 1 and 4 specify what the corrective command is, so conceptually the distal error and redundancy problems are solved, but it is worthwhile to think of how this corrective command could become associated with the DCN activity in the nervous system. We assume that Purkinje cells learn to predict the error increase, and we assume that the corresponding correction could be either an incoming efference copy (model 1), or a proprioceptive signal (model 4). How does the response of Purkinje cells leads to the correction being executed?

One approach to answer this question is to assume that the DCN cells can activate the effectors, and that the association between prediction and corrections happens in the mossy fiber to DCN synapses using the Purkinje cell inhibition both as a gating mechanism and as a learning signal. Alternatively, the association between the error prediction and the correction could happen outside the cerebellum. We elaborate on this below.

In the case of model 4, the identity of the right correction is produced at the level of the spinal cord using the equilibrium lengths from the central controller. In the case of model 1 the corrections are motor commands, so they will also be available at the spinal cord. A parsimonious hypothesis is thus that DCN activity becomes associated with corrections in the spinal cord through temporally asymmetric Hebbian learning. This hypothesis thus leads to a model where a group of microcomplexes that produces the same outputs (because they use the same learning signal), but each microcomplex targets different effectors. An equivalent model is a single micromplex that targets many different effectors, but its connection with each effector can learn independently. In either case the output of a microcomplex is associated with a response only when it happens shortly before the region of the spinal cord it innervates becomes active. There are thus two conditions to create a correction: the context is associated with an error (reflected by the DCN activity), and the effector is associated with the correction (reflected by the spinal cord activity shortly thereafter).

It has been shown that perceived errors are sufficient to produce adaptation in reaching movements, so that executing the corrective motion is not necessary for improving performance (Kitazawa et al., 1995; Tseng et al., 2007). In its present form, our model 4 may not be sufficient to explain these experimental results. On the other hand, as in Fujita (2005), movement execution is not necessary to train our first model, as long as shortly after producing an error a copy of the subsequent efferent command reaches the cerebellum, even if that command is suppressed. In the hypothesis of the previous paragraph, however, the motor command reaches the spinal cord, so depending on the particulars of the temporally asymmetric Hebbian learning suppressing the command could interfere with learning.

We can mention another hypothesis of how DCN activity (that only signals the need for a correction, but not the correction) becomes associated with muscle activations. The hypothesis is that the DCN together with the brainstem and the spinal cord could act like a multilayer perceptron that associates the activity of DCN nuclei with muscle activations that reduce the error. Corrective commands like those of models 1 and 4 permit the creation of training signals. Although this hypothesis offers great computational flexibility, it is very speculative, with many possible variations, so we don't elaborate upon it.

A prediction arising from this discussion is that when using visually generated errors the plasticity at the level of the brainstem and the spinal cord may be essential for ensuring that the cerebellar corrections achieve their intended effect, at least during the development period and for the control multiple-jointed limbs. Some models assume that plasticity in the cerebellum is distributed between the cerebellar cortex and the deep cerebellar nuclei (Raymond et al., 1996; Garrido et al., 2013). We posit one further memory site outside of the cerebellum, responsible for adjusting the effect of its outputs. The outputs of cerebellar cortex could both modulate and act as a learning signal for the vestibulum/cerebellar nuclei, while in turn the output from the cerebellar nuclei could modulate and train the response in the brainstem/spinal cord.

### 4.4. Comparison with other models

A model that is related to the model 1 in this paper was presented by Fujita (2005). In this model, associative learning is used to link motor commands with the subsequent corrections performed by a high-level controller. Fujita assumed that if a high-level motor center unit made a projection to a microcomplex, then the nuclear cells of that microcomplex and the motor center unit would encode the same information. We have no high-level motor center units in our model; instead we have searched for ways to specifically solve the distal error problem. Another difference with our model is that the context we associate with a correction may contain afferent information (e.g., Ghelarducci et al., 1975; Holtzman et al., 2006; Casabona et al., 2010), and allows for the possibility that the same motor command may require different corrections under different circumstances.

Feedback-error learning (Kawato and Gomi, 1992) is a very influential model, whose main idea (as illustrated in Figure 1B) is to use the output of a feedback controller as the learning signal for an inverse model. Some of its difficulties were mentioned in the Introduction, including the motor error problem. Considering that a feedback controller acts like a linear transformation from sensory to motor coordinates, the error signal we use in models 1 and 4 (Figures 5, 11) is similar to the error signal in motor coordinates presented in Kawato and Gomi (1992) in that it can arise due to error rising in a feedback control system, but using sensory coordinates. These sensory coordinates, being part of the control loop, are linearly related to the motor coordinates, as the feedback controller is usually a linear transformation from sensory to motor coordinates. This is consistent with the fact that both sensory and motor information is present in complex spikes (Kobayashi et al., 1998; Winkelman and Frens, 2006).

The learning signal in the recurrent architecture (Porrill and Dean, 2007) shown in Figure 1A is of a different kind, as it is related not directly to the control performance, but to the prediction errors in a forward model. The forward model in Figure 1A is predicting the response of the controlled object, whereas the forward model in Figure 1C is predicting the response of the central controller. It should be noted that cerebellar outputs not only target brainstem and spinal cord neurons, but also thalamic nuclei that convey their signals to the cerebral cortex. In this sense the cerebellar outputs could conceivably be added to both the input and output signals of a central controller, and how those outputs are used depend on the target structure and its plasticity mechanisms. It is thus possible that architectures where the cerebellar output is directed at the input of a brainstem controller—such as the recurrent architecture—could coexist with architectures where the output is added to the motor commands, such as the architecture in this paper.

An advantage of the models in this paper with respect to the recurrent architecture is that it is clear how to deal with dynamic control of 3D reaching using a multidimensional error signal (distance between hand and target). An assumption of the recurrent architecture is that the motor commands have enough information to determine the appropriate correction if a sensory error (complex spikes) arises, but this may not always be the case. For example, in the case of kinematics, the motor command completely determines the arm configuration, so the recurrent architecture is a good choice (Porrill and Dean, 2007). On the other hand, in the case of 3-dimensional arm dynamics the arm could be in any configuration after the motor command, depending on its current position and momenta. A cerebellar module receiving only muscle stimulations as its input may not have enough information to decide whether to participate in a correction when the hand gets away from the target. It would thus be beneficial to investigate how the basic recurrent architecture could improve 3-dimensional reaching when the error signals don't have a clear correlation with the motor commands. The elegant and concise structure of the basic recurrent architecture does not need any structures outside of the cerebellar cortex in order to associate errors with corrections (unlike the models in this paper), so it would be interesting to find the range of problems it can solve.

Notice that the architecture in Figure 1C, by virtue of being a forward model that uses sensory errors together with a feedback controller is compatible with simple spikes encoding sensory errors with both a lead (the future corrections associated with contexts) and a lag with the opposite modulation (the sensory error and its associated context is an input to Purkinje cells) (Popa et al., 2012, 2013, 2014). It is not clear whether this would be the case in the recurrent architecture of Figure 1A, since the input and output of the forward model (motor commands and predicted trajectories, respectively) may or may not be associated with sensory errors (sensory errors would be directly associated with complex spikes).

There are some recent models that specifically address the role of the cerebellum in reaching tasks, but for the most part they are not concerned with the distal error and redundancy problems. Some examples are presented next.

In Carrillo et al. (2008) a relatively realistic cerebellar spiking network was implemented for real-time control of a 2 DOF robot arm. The arm used open-loop control based on calculating a minimum-jerk trajectory that was transformed into a trajectory in joint angle coordinates, from which crude torque commands were generated. The cerebellum was capable of reducing the error by providing corrective torques. The redundancy problem does not arise in this context because their 2 DOF arm moves in a plane, and the elbow joint does not reach negative angles. Also, the distal error problem is not addressed since the error of their 4 microzones, each corresponding to a muscle, is provided by the Inferior Olivary (IO) input based on the difference between the desired and actual trajectories. Because of the low IO firing rates, a probabilistic encoding has to be used in order to communicate this error.

Garrido et al. (2013) used a cerebellar inverse model to implement adaptable gain control for a simulated robot arm with 3 DOF performing a smooth pursuit task. Their model used plasticity at 3 synaptic sites to produce corrective torques at states that correlated with errors. To represent states Garrido et al. used a granule cell layer model that generated sequences of binary vectors in discrete time when presented with a fixed mossy fibre pattern. The activity at Purkinje cells and DCN cells were represented with scalar values. To solve the distal error and redundancy problems this model is provided with desired trajectories in intrinsic coordinates. The difference between desired and actual trajectories is used to calculate errors in each joint by an IO module, which represents this error as a scalar value.

In another model (Casellato et al., 2014) a spiking cerebellar network was used to implement adaptive control in a real robot. Their model implemented Pavlovian conditioning, as well as adaptation in the vestibulo-ocular reflex, and in perturbed reaching. The redundancy problem and the distal error problem are not addressed, since their model only controls 1-dimensional responses.

### 4.5. Hierarchical control

An interesting aspect of our architecture comes from its application to hierarchical models such as Threshold Control Theory (TCT) (Feldman and Levin, 2009; Latash et al., 2010), and Perceptual Control Theory (PCT) (Powers, 1973, 2005). Briefly, TCT posits that movement control begins by setting a threshold value for muscle lengths. Muscle contraction happens in response to the muscle length exceeding this threshold. For a given set of threshold values, interaction with the environment brings the organism to an equilibrium position; the organism needs to learn the threshold values that result in desired equilibrium positions. To solve redundancy problems with minimal action, this paradigm can be extended hierarchically. For example, if there is a neuron that responds montonically to the aperture of the elbow angle, a controller can set a threshold value for that neuron (the neuron responds only when the elbow angle goes beyond the threshold). The elbow angle neuron can in turn set the threshold lengths of the biceps and triceps brachii muscles so that the its threshold value can in fact control the elbow angle. At a higher level, there could be neurons that respond to the arm configuration, and affect the threshold levels for neurons responding to shoulder, elbow, and wrist angles. Each hierarchical level works as a feedback control system whose set point is specified by the level above. In this paradigm, known as cascade control, each level isolates the levels above from disturbances (as long as the lower levels are on a faster timescale than the higher levels), and redundancies are resolved automatically. PCT shares some of the same ideas as TCT. In PCT the organism seeks to control its perceptions (instead of TCT's equilibrium positions), and this is achieved through a cascade control scheme, going from individual muscles to advanced cognitive operations. PCT also proposes a mechanism allowing such a hierarchy of control systems to arise.

**Figure 13:**
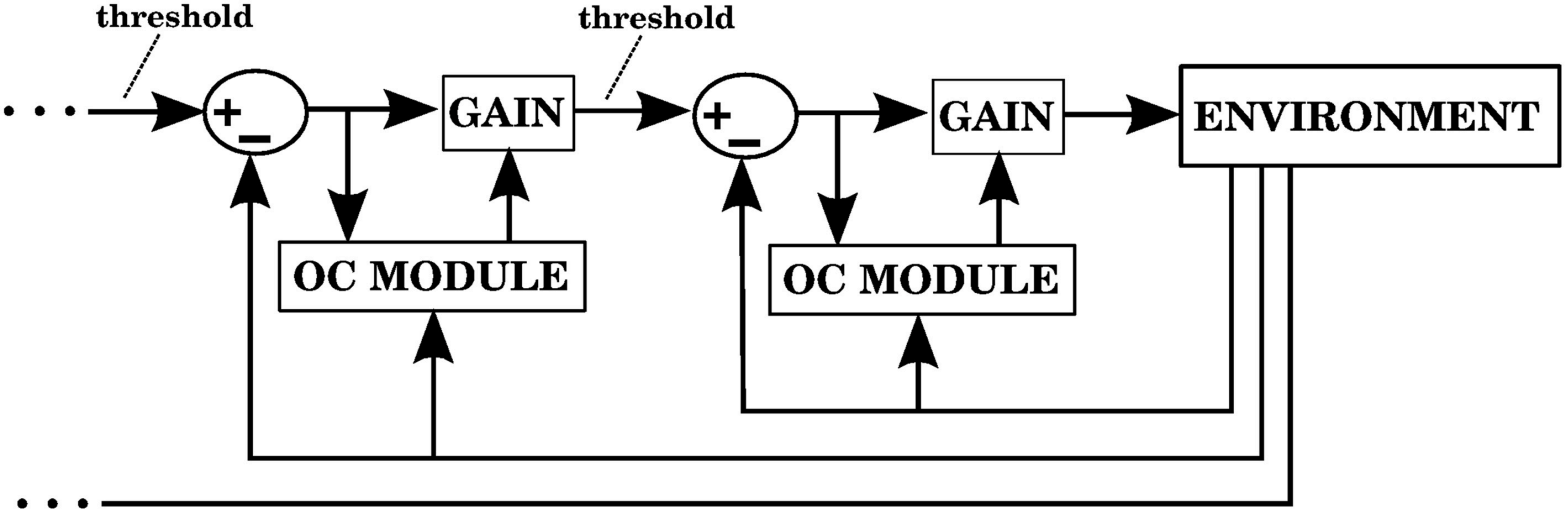
**Olivo-cerebellar modules used to anticipatively adjust threshold values in a cascade control scheme**. The difference between a received threshold value and a value perceived from the environment is transmitted to the olivo-cerebellar module. Increases in this difference cause the olivo-cerebellar module (OC-MODULE) to associate the perceived context at the time of the increase with an anticipative correction. The effect of this correction could be additive, or it could modify a gain on the signal at the GAIN block. Notice that the difference between a threshold value and a perceived value could set the threshold of more than one control loop.

Despite their advantages, TCT and PCT rely on feedback control, which can encounter problems in the presence of time delays and low gains. The cerebellar architecture presented in this paper, based on predicting the increase in error, is well suited to improve the performance of TCT or PCT models. The ideas presented in this paper offer several options to do this. Perhaps the simplest one is to generate an error signal whenever a threshold value is being exceeded (Figure 13), similarly to our model 2. The emission of this error signal can be conditioned on the error increasing on a higher level, similarly to our model 3. Or similarly to our model 4, the error signal can have its origin on a level high in the hierarcy, but the corrective signals can be generated at the lower levels using their own threshold values. This consitutes a hypothesis of how the cerebellum could function to improve motor and cognitive operations using repetitions of the same modular circuit.

### Conflict of interest statement

Randall C. O'Reilly is CTO at eCortex, Inc., which may derive indirect benefit from the work presented here. The authors declare that the research was conducted in the absence of any commercial or financial relationships that could be construed as a potential conflict of interest.
