# Supplementary material for: How the credit assignment problems in motor control could be solved after the cerebellum predicts increases in error
Source: Front Comput Neurosci. 2015 Mar 24;9:39. doi: 10.3389/fncom.2015.00039 (PMC4371707; doi:10.3389/fncom.2015.00039)
Supplement: Supplementary file 1 [file Presentation1.PDF]

Frontiers in Computational Neuroscience

Hypothesis and Theory
13 March 2015

Supplementary Material: How the credit assignment problems in motor control could be solved after cerebellum predicts increases in error

Sergio O. Verduzco-Flores [1,*], Randall C. O'Reilly [1]

[1] *Computational Cognitive Neuroscience Laboratory, University of Colorado Boulder, Department of Psychology and Neuroscience, Boulder, CO, USA*

Correspondence*:
Sergio O. Verduzco-Flores
Computational Cognitive Neuroscience Laboratory, University of Colorado Boulder, Department of Psychology and Neuroscience, 345 UCB, Boulder, CO, 80309, USA, sergio.verduzco@gmail.com

1 A FORMAL PROOF OF THE CEREBELLUM'S ABILITY TO REDUCE ERROR

Consider a point of mass m, moving under the influence of a central force $\mathbf{F}(r)$, where $r = \|\mathbf{r}\|$, $\mathbf{r}$ is the position vector of the mass, and at any moment $\mathbf{F}(r)$ is a vector directed opposite to $\mathbf{r}$ (pointing towards the origin). Define $f(r) \equiv \|\mathbf{F}(r)\|$. We assume that $f(0) = 0$, and $f'(r) > 0 \; \forall r \geq 0$.

We will identify the point mass with the hand, the origin with the target, and the force with the central controller. Before introducing a description for the idealized cerebellum we will summarize some classic results about central forces in the next lemma (Strogatz, 2001; Morin, 2008).

LEMMA 1. *Under the exclusive action of the central force, the trajectory of the point mass is: a) Contained in a plane. b) A level curve of the energy function. c) A closed curve.*

PROOF. To see that the trajectory is contained on a plane, first notice that since the force always points towards the origin, there is no torque with respect to it. By conservation of angular momentum, the angular momentum $\mathbf{L}$ of the particle is a constant vector. Let $\mathbf{p}$ denote the momentum of the particle. By definition $\mathbf{L} = \mathbf{r} \times \mathbf{p}$. This means that $\mathbf{r}$ is orthogonal to $\mathbf{L}$, but $\mathbf{L}$ is constant, so the particle moves on a plane.

At this point we can define a pair of fixed coordinate axes $\mathbf{x}$,$\mathbf{y}$ in the plane of motion. We can also define radial and tangent vectors $\mathbf{e_r}(t)$, and $\mathbf{e}_\theta(t)$, where $\mathbf{e_r}(t)$, is a unit vector pointing from the origin to the point mass at time t, and $\mathbf{e}_\theta(t)$ is a unit vector orthogonal to $\mathbf{e_r}(t)$ as in panel A of figure 1. The position of the mass is determined by its polar coordinates (r, θ), where $\theta(t)$ is the angle between $\mathbf{e_r}(t)$ and $\mathbf{x}$. The location of the system in state space is fully determined by the vector $(r, \theta, \dot{r}, \dot{\theta})$.

To prove the next two parts of the lemma, let's write down the equations of motion for the particle. The force creates a potential $V(r)$, so that $V'(r) = f(r)$. The Lagrangian of the system is

$$\mathcal{L} = \frac{1}{2} m(\dot{r}^2 + r^2\dot{\theta}^2) - V(r). \tag{1}$$

The resulting equations of motion are

$$m\ddot{r} = mr\dot{\theta}^2 - f(r), \tag{2}$$

$$\frac{d}{dt}(mr^2\dot{\theta}) = 0. \tag{3}$$

Equation 3 states the conservation of angular momentum. We can rewrite it as

$$L \equiv mr^2\dot{\theta}, \tag{4}$$

and notice that $|L| = \|\mathbf{L}\|$.

Using equation 4 to eliminate $\dot{\theta}$ from equation 2, multiplying by $\dot{r}$, and integrating, we obtain

$$E = \frac{1}{2}m\dot{r}^2 + \left(\frac{L^2}{2mr^2} + V(r)\right), \tag{5}$$

where E is a constant of integration that equals the energy of the system (the right-hand side of the equation is just the energy equation with $\dot{\theta}$ removed using equation 4). The fact that the motion of the particle obeys this equation proves the second part of the lemma.

The expression enclosed by parentheses in equation 5 is known as the effective potential.

$$V_{eff} \equiv \frac{L^2}{2mr^2} + V(r). \tag{6}$$

Since we assumed $f'(r) > 0$, $V(r)$ is a convex function, and the effective potential has the general shape shown in figure 1. The effective potential may attain the value E in at most two points r_1, r_2, which denote the minimum and maximum radii of the trajectory respectively. Notice that smaller values of E will tend to reduce r_2, and smaller values of L will reduce r_1. This will become relevant in the following theorems.

To show that the trajectory is a closed curve we use the result that in conservative systems, trajectories around isolated fixed points are closed. If we write equation 2 as two first-order differential equations, eliminate $\dot{\theta}$ using equation 4, and equate to zero we can see that the only fixed point of our system occurs at $\dot{r} = 0, f(r) = L^2/mr^3$. This is the case of circular motion.

We will now describe the idealized cerebellum, which will act by applying an instantaneous impulse to the mass whenever the current point in state space reaches one of a set of previously stored points. Each *correction* will thus consist of a pair $(\mathbf{x_c}, \mathbf{I_c})$, where $\mathbf{x_c}$ is the point of state space where the impulse $\mathbf{I_c}$ is applied.

Definition 1. Let $S(t)$ denote the point in state space of our central force system at time t. Let τ, α, d be positive real numbers, with $\alpha \in (0, 1)$. A cerebellum with speed threshold τ, displacement threshold d, and gain α will create a correction at the point $\mathbf{x_c} = S(t_c)$ at time t_c whenever two conditions are met:

1. $\dot{r}(t_c) = \tau$, and $\exists\ \xi_1 > 0$ such that $t \in (t_c - \xi_1, t_c) \Rightarrow \dot{r}(t) < \tau$.
2. $\exists\ \xi_2 > 0$ such that $\int_{t_c}^{t_c+\xi_2} \dot{r}(t)dt \geq d$, and $t \in (t_c, t_c + \xi_2) \Rightarrow \dot{r}(t) \geq \tau$.

The impulse vector to be applied at point $\mathbf{x_c}$ is obtained from:

$$\mathbf{I_c} = \alpha \int_{t_c}^{t_c+\xi_3} \mathbf{F}(r(t))dt. \tag{7}$$

The value ξ_3 in equation 7 is the least upper bound of all values ξ_3^* satisfying the next 3 conditions:

1. $t \in [t_c, t_c + \xi_3^*] \Rightarrow \dot{r}(t) \geq \tau$,

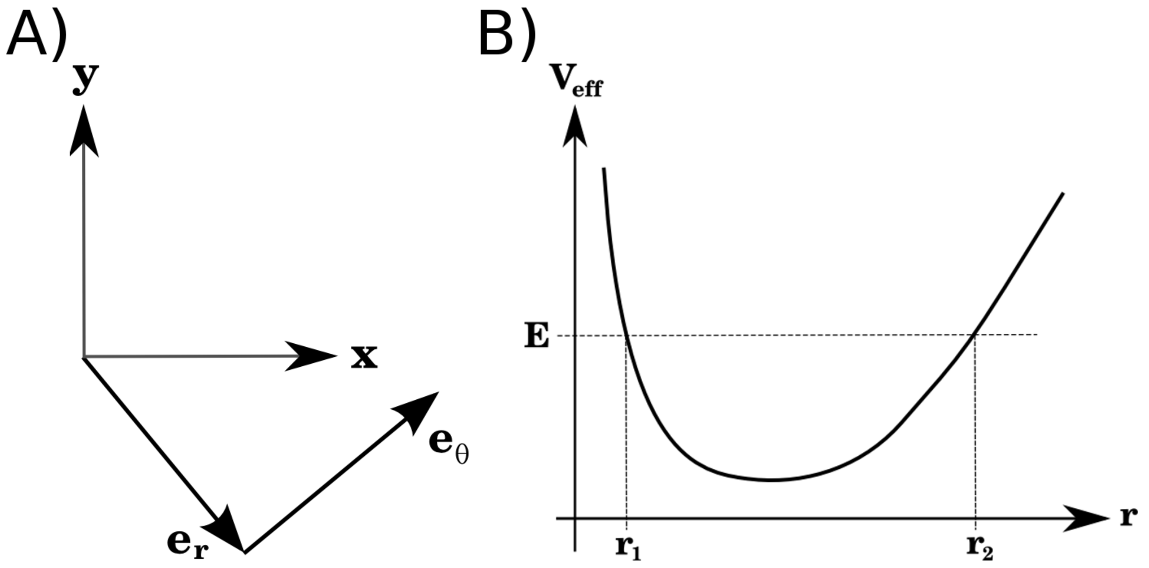

Figure 1. A) Coordinate axes for the planar motion. B) Effective potential as a function of distance from the origin

2. $|\theta(t_c + \xi_3^*) - \theta(t_c)| < \frac{\pi}{2}$,
3. $\left\| \int_{t_c}^{t_c+\xi_3^*} \mathbf{F}(r(t))dt \right\| < 2m\dot{r}(t_c)$.

The first condition for ξ_3 states that the error should be increasing at a rate of at least τ during the integration interval. The second condition for ξ_3 is that the mass shouldn't rotate around the origin more than $\pi/2$ radians. When $L = 0$ this is trivially satisfied, since equation 4 implies $\dot{\theta}(t) = 0 \; \forall t$. When $L \neq 0$ it suffices to have:

$$\xi_3^* < \frac{mr^2(t_c)\pi}{2|L|}. \tag{8}$$

In order to see this, notice that equation 4 implies $\dot{\theta} = L/mr^2$. Therefore, the condition is equivalent to $\int_{t_c}^{t_c+\xi_3^*} |L|/mr^2(t)dt < \pi/2$. But $r(t)$ is growing, so $\int_{t_c}^{t_c+\xi_3^*} |L|/mr^2(t)dt < |L|\xi_3^*/mr^2(t_c)$. Thus, a sufficient condition is $|L|\xi_3^*/mr^2(t_c) < \pi/2$. Because of this condition, in our computational simulations we reduced the probability of storing a correction when the value of r was small. The third condition for ξ_3 means that the impulse will not be powerful enough to increase the original magnitude of the radial velocity after reversing it.

Applying an instantaneous impulse is akin to "teleporting" to a different point of state space, and is physically impossible. This abstraction models the application of an anticipative correction close to the point of "teleportation", in which case the impulse is applied through a force that is active for a short period of time.

We are now ready to prove that in the case of repeated identical trajectories the cerebellum reduces the energy and angular momentum of the system.

THEOREM 1. *Let the trajectory of the system in state space $S(t)$ have initial conditions $S_0 = (r_0, \dot{r}_0, \theta_0, \dot{\theta}_0)$ with $r_0 > 0$, and assume that a correction is created at point $\mathbf{x_c} = S(t_c)$. If we start the trajectory once more at S_0, and apply the correction at $\mathbf{x_c}$, then the energy E of equation 5 will be reduced after the impulse is applied. If $L > 0$, the angular momentum will also be reduced.*

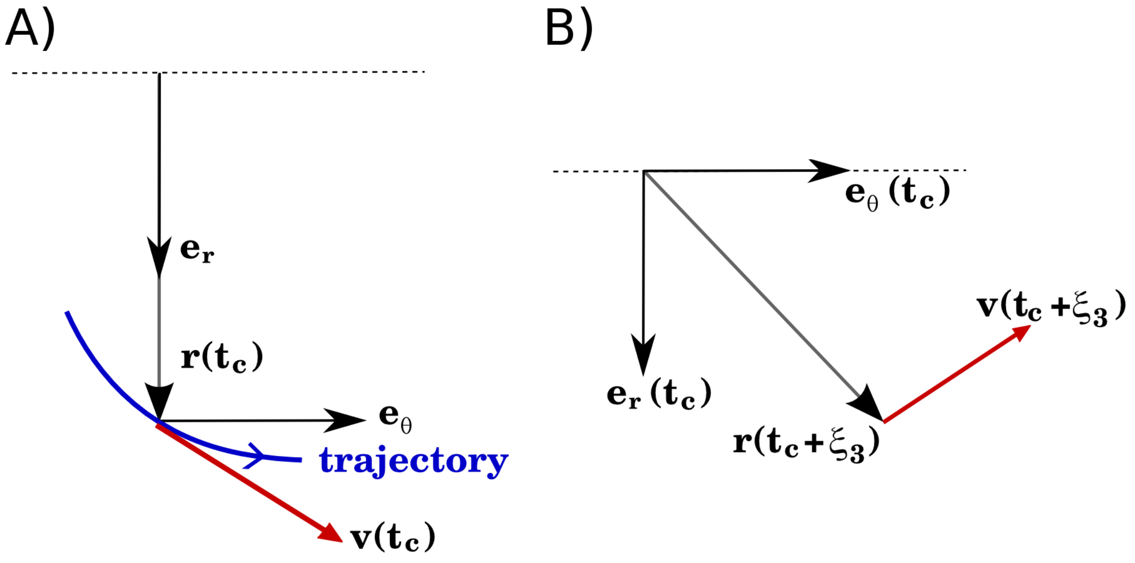

Figure 2. A) A rotated view of the motion, where the vector $\mathbf{e_r}(t_c)$ points downwards and the vector $\mathbf{e}_\theta(t_c)$ points to the right. B) General direction of all vectors when the correction is formed.

PROOF. For visualization purposes, we will take advantage that save for a reflection, the trajectory of the point mass at time t_c will always look as in figure 2A. Write the correction's impulse as $\mathbf{I_c} = I_r\mathbf{e_r}(t_c) + I_\theta\mathbf{e}_\theta(t_c)$, and the particle's momentum as $\mathbf{p}(t_c) = m\mathbf{v}(t_c) = mv_r(t_c)\mathbf{e_r}(t_c) + mv_\theta(t_c)\mathbf{e}_\theta(t_c)$. Notice that $L = mv_\theta(t)$, and $\dot{r}(t) = v_r(t)$.

We first show that $\mathbf{I_c}$ acts to reduce $|L|$. To do this, we show that $I_\theta v_\theta \leq 0$, and $|I_\theta| < |mv_\theta|$. Since the momentum $\mathbf{p}$ after the application of the impulse comes from the previous momentum plus $\mathbf{I_c}$, then L will be reduced.

Assume that integration in equation 7 stops due to condition 1. Then the velocity of the particle at time $t_c + \xi_3$ will be almost tangential to $\mathbf{e_r}(t_c + \xi_3)$, but pointing a bit "to the right" depending on the value of τ (figure 2B), which means a positive $\mathbf{e}_\theta(t_c)$ projection. $\mathbf{e_r}(t_c + \xi_3)$ will be in between $\mathbf{e_r}(t_c)$ and $\mathbf{e}_\theta(t_c)$, otherwise condition 2 would be false. Therefore, in the interval $(t_c, t_c + \xi_3)$ $\mathbf{F}(r(t))$ points to the left and $\mathbf{v}(t)$ points to the right, so $I_\theta v_\theta < 0$. Moreover, the accumulated impulse from integrating $\mathbf{F}(r(t))$ is not sufficient to make $\mathbf{v}(t)$ point to the left, so $|I_\theta| < m|v_\theta(t_c)|$.

If integration in equation 7 stops due to condition 2, this means that the particle is still moving "to the right" when crossing the horizontal line going through the origin, as otherwise condition 1 would be false. The same argument as in the previous case is applicable.

If the integration stops due to condition 3, then the velocity will still point to the right, since equation 4 implies that the angle increases monotonically.

To show that the energy is decreased after the impulse, we will show that the kinetic energy component $(1/2)m\dot{r}^2$ in equation 5 is reduced. Since the effective potential only depends on the radius, and the impulse does not change the radius at the moment of its application, then the energy will decrease.

From condition 2 we know that $\mathbf{F}(r(t))$ has a positive upwards component in $(t_c, t_c + \xi_3)$, so $I_r v_r(t_c) < 0$. Condition 3 ensures that even if the impulse reverses the direction of v_r, its magnitude will not increase; namely $|I_r| < 2m|v_r(t_c)|$. Therefore $\dot{r}^2$ will decrease its value, and this is supported by the fact that $\alpha < 1$.

Theorem 1 shows that the cerebellum works well when encountering error-prone points of state space that were visited before. These are discrete points, however, and form a set of measure zero (i.e. a set with no volume). If we expect the cerebellum to generalize its corrections to some extent, then those corrections should still be useful when applied at points of state space near the point where the correction was created. The next theorem addresses this issue.

THEOREM 2. *Let $S(t)$ denote an arbitrary trajectory of the system through state space. Assume the cerebellum has stored a correction $(\mathbf{x_c}, \mathbf{I_c})$. Then $\exists\ \eta > 0$ such that $\|S(t^*) - \mathbf{x_c}\| < \eta$ implies that $\mathbf{I_c}$ applied at $S(t^*)$ will reduce the energy of the system.*

PROOF. Let's write $\mathbf{x_c} = (r_c, \dot{r}_c, \theta_c, \dot{\theta}_c)$. Let $\mathbf{e_r}(t_c)$ and $\mathbf{e}_\theta(t_c)$ be the radial and tangential vectors corresponding to the point $\mathbf{x_c}$. The impulse of the correction and the velocity of the point at t_c in the trajectory where the correction was created can be written as
$\mathbf{I_c} = I_r \mathbf{e_r}(t_c) + I_\theta \mathbf{e}_\theta(t_c)$,
$\mathbf{v_c} = v_r(t_c)\mathbf{e_r}(t_c) + v_\theta \mathbf{e}_\theta(t_c) = \dot{r}_c \mathbf{e_r}(t_c) + (L/m)\mathbf{e}_\theta(t_c)$.

Right after the impulse application at $\mathbf{x_c}$ the new radial velocity will be $\dot{r}_c^+ = \dot{r}_c + (I_r/m)$, and the new angular momentum will be $L^+ = L + I_\theta$. The change in energy after the impulse will thus be:

$$\Delta E_c \equiv \frac{1}{2}m\left(\dot{r}_c + (I_r/m)\right)^2 + \frac{(L+I_\theta)^2}{2mr_c^2} - \frac{1}{2}m\dot{r}_c^2 - \frac{L^2}{2mr_c^2}$$
$$= \dot{r}_c I_r + \frac{I_r^2}{2m} + \frac{I_\theta}{mr_c^2}(L + I_\theta/2). \tag{9}$$

Let θ_I be the angle between the $\mathbf{I_c}$ vector and the $\mathbf{x}$ axis. The angle ϕ_c formed by the vectors $\mathbf{I_c}$ and $\mathbf{e_r}(t_c)$ can be written as $\phi_c = \theta_I - \theta_c$. We may also write $I_r = \|\mathbf{I_c}\|\cos(\phi_c)$, $I_\theta = \|\mathbf{I_c}\|\sin(\phi_c)$. Substituting this and equation 4 into equation 9 we get

$$\Delta E_c = \|\mathbf{I_c}\|\dot{r}_c\cos(\phi_c) + \frac{\|\mathbf{I_c}\|^2\cos^2(\phi_c)}{2m} + \|\mathbf{I_c}\|\sin(\phi_c)\left(\dot{\theta}_c + \frac{\|\mathbf{I_c}\|\sin(\phi_c)}{2mr_c^2}\right). \tag{10}$$

Using equation 10 we may explore how the energy changes as a function of where the impulse is applied in state space. For a given vector $\mathbf{s} = (r, \dot{r}, \theta, \dot{\theta})$ with $r > 0$ we may define a function

$$\Delta E(\mathbf{s}) \equiv \|\mathbf{I_c}\|\dot{r}\cos(\phi) + \frac{\|\mathbf{I_c}\|^2\cos^2(\phi)}{2m} + \|\mathbf{I_c}\|\sin(\phi)\left(\dot{\theta} + \frac{\|\mathbf{I_c}\|\sin(\phi)}{2mr^2}\right), \tag{11}$$

where $\phi = \theta_I - \theta$. From theorem 1 we know that $\Delta E(\mathbf{x_c}) = \Delta E_c < 0$. We want to know if there is a sphere centered at $\mathbf{x_c}$ where all values of ΔE are negative. To do this, we will use the fact that the gradient

of ΔE is bounded near $\mathbf{x_c}$. We'll start by obtaining the partial derivatives.

$$\begin{aligned}
\frac{\partial \Delta E}{\partial r} &= -\frac{\|\mathbf{I_c}\|^2 \sin^2(\phi)}{mr^3}, \\
\frac{\partial \Delta E}{\partial \dot{r}} &= \|\mathbf{I_c}\| \cos(\phi), \\
\frac{\partial \Delta E}{\partial \theta} &= -\frac{\partial \Delta E}{\partial \phi} \\
&= \|\mathbf{I_c}\| \left(\dot{r}\sin(\phi) - \dot{\theta}\cos(\phi) + \frac{\|\mathbf{I_c}\| \cos(\phi)\sin(\phi)}{m} \left(1 - 1/r^2\right) \right), \\
\frac{\partial \Delta E}{\partial \dot{\theta}} &= \|\mathbf{I_c}\| \sin(\phi).
\end{aligned}$$

Notice that $r_c \geq d > 0$. Let $\delta_1 = \frac{r_c}{2}$, and define the ball $B(\mathbf{x_c}, \delta_1) \equiv \{\mathbf{s} \in \mathbb{R}^4 | \; \|\mathbf{x_c} - \mathbf{s}\| < \delta_1\}$. It is easy to see that if $\mathbf{s} \in B(\mathbf{x_c}, \delta_1)$ then

$$\begin{aligned}
\left| \frac{\partial \Delta E}{\partial r}(\mathbf{s}) \right| &\leq \frac{8\|\mathbf{I_c}\|^2}{mr_c^3} \equiv h_1, \\
\left| \frac{\partial \Delta E}{\partial \dot{r}}(\mathbf{s}) \right| &\leq \|\mathbf{I_c}\|, \\
\left| \frac{\partial \Delta E}{\partial \theta}(\mathbf{s}) \right| &\leq \|\mathbf{I_c}\| \left(\dot{r}_c + |\dot{\theta}_c| + r_c + \frac{2\|\mathbf{I_c}\|}{m} \max\left\{ \frac{1}{4}, \frac{1}{r_c^2} \right\} \right) \equiv h_2, \\
\left| \frac{\partial \Delta E}{\partial \dot{\theta}}(\mathbf{s}) \right| &\leq \|\mathbf{I_c}\|.
\end{aligned}$$

The Fundamental Theorem of Calculus for line integrals states that for any given trajectory γ of length λ starting at $\mathbf{x_c}$ and ending at some point $\mathbf{s_B}$ on the boundary of $B(\mathbf{x_c}, \delta_1)$, and with tangent vector $\nu(t)$ we have

$$\Delta E(\mathbf{s_B}) - \Delta E_c = \int_\gamma \nabla(\Delta E) \cdot d\nu.$$

We define the number $\mathbb{J}$ to be the magnitude of the vector $(h_1, \|\mathbf{I_c}\|, h_2, |\mathbf{I_c}\|)$. From the Cauchy-Schwarz inequality we have

$$\int_\gamma \nabla(\Delta E) \cdot d\nu \leq \mathbb{J}\lambda.$$

Since only the end points of the trajectory determine the value of the integral, we may choose a straight line from $\mathbf{x_c}$ to the boundary of $B(\mathbf{x_c}, \delta_1)$, yielding

$$\int_\gamma \nabla(\Delta E) \cdot d\nu \leq \mathbb{J}\delta_1. \tag{12}$$

Considering that $\Delta E(\mathbf{s_B}) = \Delta E_c + \int_\gamma \nabla(\Delta E) \cdot d\nu$, we can ensure that $\Delta E(\mathbf{s_B})$ remains negative if we make a short enough displacement along the straight line trajectory of equation 12. Namely, we can find a displacement δ_2 such that $\mathbb{J}\delta_2 < |\Delta E_c|$. Take

$$\eta = \min\{\delta_1, \delta_2\}.$$

We can now ensure that if $\mathbf{s} \in B(\mathbf{x_c}, \eta)$ then $\Delta E(\mathbf{s}) < 0$.

One final issue to address is that the two theorems above assume that the only force acting on the point mass is the force field $\mathbf{F}(r)$. This ceases to be true when we start storing corrections associated with balls of positive radius in state space, since these corrections provide an impulse to the mass. When these balls don't overlap it is clear that the conclusions of the theorems are still valid. We could then modify the radii of the balls so that they don't overlap. In practice we don't find a necessity of ensuring that zones associated with a correction don't overlap in our computational model.

REFERENCES

Strogatz, S. H., *Nonlinear Dynamics And Chaos: With Applications To Physics, Biology, Chemistry, And Engineering* (Westview Press, 2001), 1st edition.

Morin, D., *Introduction to Classical Mechanics: With Problems and Solutions* (Cambridge University Press, 2008).
